# Paeoniflorin directly binds to TNFR1 to regulate podocyte necroptosis in diabetic kidney disease

**DOI:** 10.3389/fphar.2022.966645

**Published:** 2022-09-06

**Authors:** Xian Wang, Xue-qi Liu, Ling Jiang, Yue-bo Huang, Han-xu Zeng, Qi-jin Zhu, Xiang-ming Qi, Yong-gui Wu

**Affiliations:** ^1^ Department of Nephropathy, The First Affiliated Hospital of Anhui Medical University, Hefei, China; ^2^ Center for Scientific Research of Anhui Medical University, Hefei, China

**Keywords:** paeoniflorin, tumor necrosis factor receptor 1, podocyte, necroptosis, diabetic kidney disease

## Abstract

Necroptosis was elevated in both tubulointerstitial and glomerular renal tissue in patients with diabetic kidney disease (DKD), and was most pronounced on glomerulus in the stage with macroalbuminuria. This study further explored whether paeoniflorin (PF) could affect podocyte necroptosis to protect kidney injure *in vivo* and *in vitro*. Our study firstly verified that there are obvious necroptosis-related changes in the glomeruli of DKD through bioinformatics analysis combined with clinicopathological data. STZ-induced mouse diabetes model and high-glucose induced podocyte injury model were used to evaluate the renoprotection, podocyte injury protection and necroptosis regulation of PF in DKD. Subsequently, the target protein-TNFR1 that PF acted on podocytes was found by computer target prediction, and then molecular docking and Surface plasmon resonance (SPR) experiments were performed to verify that PF had the ability to directly bind to TNFR1 protein. Finally, knockdown of TNFR1 on podocytes *in vitro* verified that PF mainly regulated the programmed necrosis of podocytes induced by high glucose through TNFR1. In conclusion, PF can directly bind and promote the degradation of TNFR1 in podocytes and then regulate the RIPK1/RIPK3 signaling pathway to affect necroptosis, thus preventing podocyte injury in DKD. Thus, TNFR1 may be used as a new potential target to treat DKD.

## 1 Introduction

Diabetic kidney disease (DKD) has been the major cause of chronic kidney disease replacing chronic glomerulonephritis in Chinese inpatients (Zhang et al., 2016). An early manifestation of DKD includes microalbuminuria, which is closely related to the damage to the glomerular filtration barrier (GFB). Moreover, to podocytes injury (loss of functions and reduced number), as a key component of GFB, is considered a major contributor to DKD (Zhang et al., 2020). Therefore, studying the mechanism of podocyte injury may provide a basis for targeted prevention and treatment of DKD.

Transdifferentiation and death were the ultimate outcome of cell damage. Epithelial-mesenchymal transition (EMT) and endothelial-mesenchymal transition (endMT) involve in the renal fibrosis process of DKD (Zeisberg et al., 2003; Zeisberg et al., 2008; Zeng et al., 2019; Chen et al., 2022). Interestingly, markers of mesenchymal cells were also found on podocytes in DKD, suggesting that podocytes also exhibit EMT (Li et al., 2008), which may be regulated by multiple signaling pathways, including TGF-β (Yin et al., 2018), Wnt/β-catenin (Kato et al., 2011), mTOR (Tao et al., 2021), Notch (Nishad et al., 2020), Hedgehog (Lan et al., 2017), SIRT/NF-κB (Wang et al., 2019), DPP-4 (Chang et al., 2017) and non-coding RNA network (Ling et al., 2018). Additionally, the forms of programmed cell death include apoptosis, pyroptosis, necroptosis, and ferroptosis. Apoptosis is regulated by the Bcl-2 or caspase family. Pyroptosis is Gasdermin-dependent cell death. Ferroptosis is iron-dependent and peroxidative-driven, and necroptosis is associated with membrane rupture by p-MLKL. The above forms of cell death have been reported in podocyte injury in DKD (Zhang et al., 2021; Zhu et al., 2021), while unfortunately, the research of necroptosis on podocytes in DKD is still limited to *in vitro* cells (Chung et al., 2022). Our lab recently discovered the activation of necroptosis in DKD tissues glomeruli. Therefore, antagonizing necroptosis may be a therapeutic target for DKD.

In recent years, based on the mechanism research of DKD, some potential drugs have been developed, including glucose cotransporter 2 (SGLT2) inhibitors (Tomita et al., 2020), glucagon-like peptide-1 (GLP-1) inhibitors (Moellmann et al., 2018), dipeptidyl peptidase-4 (DPP-4) inhibitors (Peng et al., 2019), protein kinase C (PKC) inhibitors (Koya et al., 2000), advanced glycation end product (AGE) inhibitors (Sugimoto et al., 2007), aldosterone receptor inhibitors (De Zeeuw et al., 2010), endothelin receptor (ETR) inhibitors (De Zeeuw et al., 2014), transforming growth factor-β (TGF-β) inhibitors (Benigni et al., 2003), Rho kinase (ROCK) inhibitors (Komers et al., 2011) and N-acetyl-seryl-aspartyl-lysyl-proline (AcSDKP) (Srivastava et al., 2020; Wang et al., 2021). However, the clinical application of these drugs in DKD still has a long way to go.

Chinese herbal medicines have been used to treat diseases in China for more than a thousand years, and the efficacy and safety of most of them have been clinically proven. Paeoniflorin (PF), as an active monomer extracted from Paeonia lactiflora, have been reported a protective effect on various organ damage caused by different diseases (Zhang and Wei, 2020). Our previous study found that PF can improve kidney injury in DKD mice model (Zhang et al., 2017). In this study, we further explored whether PF can affect the necroptosis of podocytes and protect their injury in DKD mice.

## 2 Materials and methods

### 2.1 Data collection and analysis

This study extracted the specific data information of necroptosis-related genes from three groups of samples found in the GSE142025 dataset (GPL 20301) (Fan et al., 2019) from NCBI-GEO (http://www.ncbi.nlm.nih.gov/geo/), including NC group (*n* = 9), DKD1 group (albumin to creatinine ratio, ACR 30–300 mg/g, eGFR>90 ml/min·1.73 m^2^, *n* = 6), and DKD2 group (ACR>300 mg/g, eGFR<90 ml/min·1.73 m^2^, *n* = 21; one sample in the DKD2 group was missing). The necroptosis-related genes were queried from AmiGO 2 (http://amigo.geneontology.org/amigo/).

### 2.2 Patients and specimens

Percutaneous kidney biopsy samples were obtained from patients with DKD between January 2019 and December 2021. The inclusion criteria were: 1) age ≥18 years; 2) diagnosed with diabetes as previous (American Diabetes, 2013); 3) urinary ACR >30 mg/g; 4) diagnosis of DKD by renal biopsy as previous (Tervaert et al., 2010); The exclusion criteria included infection, cancer, autoimmune disease, and severe heart failure of other organs (including heart, liver, respiratory). Additionally, the unaffected portion of tumor nephrectomies were obtained to be as the samples of NC group without diabetes (urinary ACR<30 mg/g).

The study was reviewed and approved by the institutional review board of the First Affiliated Hospital of Anhui Medical University (approval no.: 20,190,454). The participants provided their written informed consent to participate in this study.

### 2.3 Chemicals and reagents

PF (CAS No: 23,180-57-6, purity = 98.04%), necrostatin-1 (Nec-1, CAS No: 4,311-88-0, purity = 99.82%), and MG132 (CAS No.: 133,407-82-6, purity≥98.0%) were purchased from Macklin Biochemical Co., Ltd. (Shanghai, China). d-glucose, streptozotocin (STZ), and D-mannitol were acquired from Sigma-Aldrich (MO, United States). Anti-RIPK1 (receptor-interacting serine-threonine kinase 1), anti-RIPK3 (receptor-interacting serine-threonine kinase 3), anti-WT-1 (wilms Tumor 1 protein), anti-TNFR1, anti-ubiquitin, anti-β-actin and secondary antibodies were from Proteintech Group, Inc. (Hubei, China). TNFR1 protein was obtained from Abcam (Cambridge, United Kingdom). Anti-SYNPO (synaptopodin) antibody was acquired from Santa Cruz (Santa Cruz Biotechnology. United States). Anti-p-MLKL (phosphorylated mixed-lineage kinase domain-like protein) was obtained from Affinity. Additionally, an immunohistochemistry kit (PV-9000) and Mayer (ZLI-9620) were acquired from Beijing Zhongshan Biotechnology Inc. (Beijing, China). Periodic acid–Schiff (PAS) kits were purchased from Jiancheng Biotechnology Institute (Nanjing, China).

### 2.4 Animal model and experimental design

We observed the effect of the whole course or preventive treatment of PF treatment in mice models. Wild-type (WT) C57BL/6J mice (male, 6–8 W, 16–20 g) were obtained from the Animal Department of Anhui Medical University (Hefei, Anhui Province, China). STZ (50 mg/kg/mice) intraperitoneal injection was used to establish a diabetic mouse model (Liu et al., 2022).

Mouse were divided into 6 groups (*n* = 6/group): normal control (NC) group, NC + PF group (200 mg/kg·d), DKD group, and DKD + PF group (50/100/200 mg/kg·d). The treatment groups were intraperitoneally injected with PF daily, and the non-treatment groups received a 0.9% sodium chloride solution. The treatment lasted for 12 weeks. A preliminary experiment showed that DKD was significantly improved by the administration of PF100 mg/kg·d. Therefore, two additional groups were established: NC + PF100 mg/kg·d and DKD + PF100 mg/kg·d, in which the treatment was given for 6 weeks and then discontinued for the following 6 weeks after the successful production of the diabetes mice model.

Metabolic cages (Hatteras Instruments, Hatteras, NC) were used to collect animal’s urine. All animals were raised under standard conditions, and the experiment protocol has been approved by the Ethics Committee of the Animal Research of Anhui Medical University (approval no.: LLSC 20190,519).

### 2.5 Physical and biochemical analyses

Before the mice were sacrificed, mice’s tail vein blood was collected and used to detect fasting blood sugar (FBS). Mice were then weighed and the blood by retro-orbital bleeding was collected under anesthesia. Mice were then euthanized, and the kidneys of the mouse were removed. A mouse albumin ELISA kit (ab108792) (Abcam, Cambridge, United Kingdom) was used to detect 24 h urinary albumin excretion rate (24 h UAER). Orbital blood was used to measure the levels of blood urea nitrogen (BUN), serum creatinine (Scr), and serum alanine transaminase (ALT) according to the kit instructions, respectively (Nanjing Jiancheng Bioengineering Institute, Nanjing, China).

### 2.6 Kidney histological examination

Kidney tissues were routinely dehydrated, embedded in paraffin, and then cut into 2 μm-thick sections. Periodic Acid-Schiff stain (PAS) stained the tissue sections as previously described (Zhong et al., 2019).

### 2.7 Transmission electron microscopy

The changes of the basement membrane and foot process were observed under an electron microscope after routine embedding and sectioning of renal tissue, as previously described (Zhong et al., 2019). In addition, the organelles and membranes of podocytes cultured *in vitro* were observed by electron microscope (Huang et al., 2020).

### 2.8 Molecular docking

The PubChem database (https://pubchem.ncbi.nlm.nih.gov/) was used to acquire the docked compound PF, which was imported into Schrodinger software to hydrogenate, structure optimize, and minimize energy. Afterward, the adjusted molecular structures were saved as ligand molecules for molecular docking. TNF (PDB ID: 7KPA) and TNFRSF1A (tumor necrosis factor receptor superfamily, member 1, APDB ID: 1EXT) protein structures were obtained by homology modeling (Swiss-Model) from the website (https://swissmodel.expasy.org/). All protein structures were processed on Schrodinger’s Protein Preparation Wizard platform, including removal of water and ions, protonation, the addition of missing atoms and completion of missing groups, and protein energy minimization. The processing and optimization of small molecules and proteins were performed by the Glide module and Protein Preparation Wizard in the Schrödinger Maestro software, respectively. Receptors were constrained minimized through the OPLS3e force field. We used the interaction interface of TNF with TNFRSF1A to determine the active site of the two docking proteins. By analyzing the action mode of compounds and proteins, the interaction between compounds and target protein residues was obtained, including hydrophobic interaction, hydrogen bonding and π-π interaction, to evaluate the molecular binding activity of compounds.

### 2.9 Surface plasmon resonance

SPR was used to observe the real-time interaction between PF and TNFR1 using a Biacore T200 (GE, United States). All reagents used in the experiment were prepared according to the instructions. TNFR1 protein was purchased from Macklin Biochemical Co., Ltd. (Shanghai, China) at 10 μg/ml was dialyzed on a Series S Sensor Chip CM5 (10 min activation with 0.5 M EDC/0.25 M NHS in 0.1 M MES, pH 5.5) to obtain an immobilization signal of 16,131.9 RU. The experimental results were calculated for the equilibrium binding and disassociation constants.

### 2.10 Mouse podocyte clone 5 culture

MPC5 cells were purchased from the Cell Bank of the Chinese Academy of Sciences (Shanghai, China), differentiated in the environment as previously reported (Zhong et al., 2019), and then cultured in DMEM (5.5 mmol/L glucose, HyClone, United States) containing 10% fetal bovine serum (Gibco, San Diego, CA, United States). Cells were then divided into 8 groups: NC group (5.5 mmol/L glucose), M group (mannitol, 34.5 mmol/L mannitol +5.5 mmol/L glucose), NC+PF (160 μmol/L), HG (high glucose, 40 mmol/L), and HG+PF (40/80/160 μmol/L) containing 1% fetal bovine serum for 48 h. Additionally, another group was treated with HG+PF 80 μmol/L for 24 h and then without PF for 24 h to verify the effect of PF preventive treatment *in vitro*. Moreover, additional cell models were established to observe the effect of PF on podocyte necroptosis *in vitro*, including NC group (5.5 mmol/L glucose), NC + Nec-1 group (5.5 mmol/L glucose +3 μmol/L Nec-1), HG group (high glucose, 40 mmol/L glucose), HG + PF group (80 μmol/L PF), HG + Nec-1 group (3 μmol/L Nec-1), and HG+PF + Nec-1 group.

### 2.11 Determination of cell vitality

The vitality of MPC5 was determined using MTT (thiazole blue colorimetry) assay. Briefly, MPC5 were treated with HG and/or PF for 48 h, followed by 20 μL of sterile MTT dye (5 mg/ml) for another 4 h. The optical density (OD) at 550 nm wavelength was measured by a microplate reader (Multiskan MK3; Thermo Scientific, Waltham, MA, United States).

### 2.12 Western blot

The protein was extracted using RIPA buffer (Beyotime, Shanghai, China) to quantify the concentrations using the BCA Kit (Beyotime, Jiangsu, China). Podocyte-rich kidney tissues were isolated as previously described (Wei et al., 2020). The total protein from each sample (20 μg) was loaded, electrophoresed, and then transferred to nitrocellulose membranes (Invitrogen, United States). Samples were then incubated with the corresponding primary antibody: anti-WT-1 (1:1,000, 12609-1-AP), anti-SYNPO (1:1,000, sc-515842), anti-RIPK1 (1:1,000, 17519-1-AP), anti-RIPK3 (1:1,000, 17563-1-AP), anti-p-MLKL (1:1,000, AF7420), TNFR1 (1:1,000, 21574-1-AP), anti-ubiquitin (1:1,000, 10201-2-AP), anti-β-actin (1:10000, 66009-1-Ig), for at least 12 h followed by sealing with blocking buffer (EpiZyme, Shanghai, China) for 10 min, and the secondary antibody (1:10,000, anti-mouse: SA00001-1 or anti-rabbit: SA00001-2) for 1h. The images of the band were developed using SuperSignalTM West Femto Maximum Sensitivity Substrate Kit (thermos scientific, United States) and Amersham Imager 600 (GE, United States), and then analyzed using ImageJ software.

### 2.13 Ribonucleic acid isolation and real-time polymerase chain reaction

Total RNA extraction of MPC5 and kidney tissues was performed using TRIZOL lysis buffer (Invitrogen, Carlsbad, CA, United States). The concentration and purity were then measured, after which the RNA was reverse transcribed into cDNAs which were amplified by using SYBR Green ER qPCR Supermix (Thermo Fisher Scientific, Waltham, MA) on a T100 thermal cycler (Bio-Rad, Hercules, CA, United States). All primers were shown in Table 1. The target mRNAs were calculated as previously reported (Zhong et al., 2019).

**TABLE 1:** Sequences of the primers.

Genes	Forward (5–3′)	Reverse (5–3′)
Mouse TNFR1	GTG​TGG​CTG​TAA​GGA​GAA​CCA​G	CAC​ACG​GTG​TTC​TGA​GTC​TCC​T
Mouse TNF-α	GGT​GCC​TAT​GTC​TCA​GCC​TCT​T	GCC​ATA​GAA​CTG​ATG​AGA​GGG​AG
Mouse IL-1β	TGG​ACC​TTC​CAG​GAT​GAG​GAC​A	GTT​CAT​CTC​GGA​GCC​TGT​AGT​G
Mouse β-actin	CAT​TGC​TGA​CAG​GAT​GCA​GAA​GG	TGC​TGG​AAG​GTG​GAC​AGT​GAG​G

### 2.14 Co-immunoprecipitation assay

MPC5 cells were divided into 4 groups: HG group (40 mmol/L glucose), HG + MG132 group (4 μmol/L MG132), HG + PF group (80 μmol/l PF), and HG + PF + MG132 group. All MPC5 cells were cultured for 36 h in a regular incubator. After being extracted from MPC5 cells, the total protein was incubated with 15 μL of protein A/G magnetic beads (MedChenExpress, Shanghai, China) overnight at 4°C. Next, the compound was mixed with a TNFR1 antibody (10 μL/tube, 21574-1-AP) at 4°C for 12 h, cleaned by RIPA buffer and boiled at 100°C for 5 min. Finally, the levels of TNFR1 and ubiquitin of the samples were detected using WB.

### 2.15 Cell transfection

After planted in the 6-well plate, MPC5 cells were transfected with TNFR1 siRNA (Hanbio, Shanghai, China) using a LipofectamineTM 2000 reagent (Invitrogen, Carlsbad, CA, United States) at 37°C for 6 h according to the manufacturer’s instructions. Subsequently, the effect of siRNAs was measured by real-time PCR and WB.

### 2.16 Immunohistochemistry assay

After routine deparaffinization, paraffin sections were heated in a pressure pot for 2 min to retrieve the antigens, incubated with the primary antibody: anti-WT-1 (1:500, 12609-1-AP), anti-SYNPO (1:500, sc-515842), anti-p-MLKL (1:300, AF7420), TNFR1 (1:300, 21574-1-AP), at 4°C overnight and the secondary antibody at 37°C for 1 h, successively. ImageJ was used to analyze the intensity of DAB staining.

### 2.17 Immunofluorescence assay

After blocking the non-special antigens with 10% goat serum, the renal tissue sections and cell slides were incubated with the primary antibody with the same concentration in the IHC experiments, and with the fluorescent secondary antibody (1:200, SA00013-1 and SA00013-4) at 37°C for 40 min, finally stained with DAPI (G1012, Servicebio). The images were taken using a fluorescence microscope (Zeiss Spot; Carl Zeiss Ltd., Canada).

### 2.18 Statistical analysis

R3.6.3 (https://www.r-project.org/) was used for the visualization of the dataset. PASS 15.0 was used to calculate the sample size of clinical trials. SPSS 22.0 was used for data analysis. Mann-Whitney *U*-test was used to calculate the statistical significance of non-normally distributed data. The statistical significance of normally distributed data among groups was calculated by independent samples *t*-test and one-way analysis of variance (ANOVA). Linear correlation analysis was performed using Spearman’s test. A *p* < 0.05 was considered to be statistically significant. Data were calculated as the mean ± SD. A *p*-value < 0.05 among groups was identified, followed by mapping with GraphPad Prism 9.3 software (GraphPad Software Inc., San Diego, CA, United States).

## 3 Results

### 3.1 The changes of necroptosis in diabetic kidney disease

#### 3.1.1 Different necroptosis-related genes are seen in human kidney tissue with diabetic kidney disease

**FIGURE 1:**
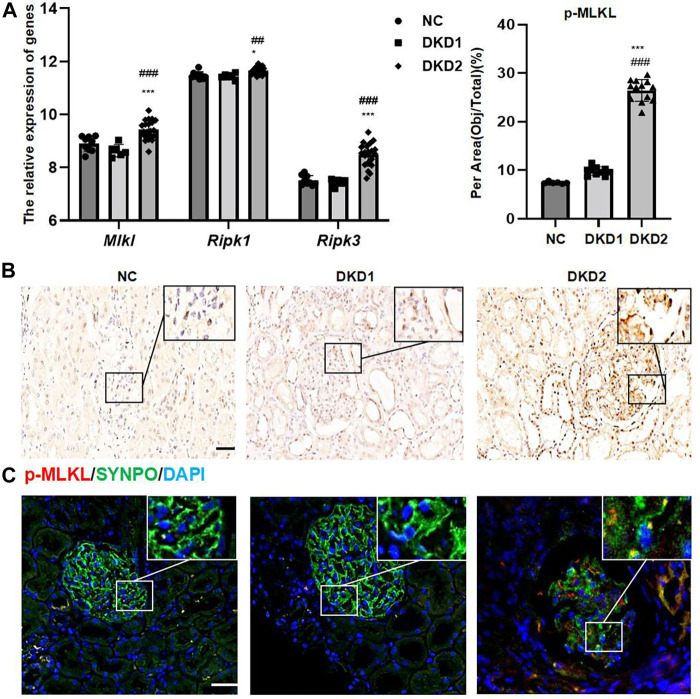
Necroptosis-related proteins changed in human kidney tissue with DKD. **(A)** The relative expression of necroptosis-related genes in GSE 142025. **(B)** IHC assay of p-MLKL protein expression in the glomerulus of human renal biopsy tissues. Scale bar = 20 μm. **(C)** IF double staining for p-MLKL and SYNPO in human renal biopsy tissues. Scale bar = 20 μm. Results represent the mean ± SD. ^*^ *p* < 0.05, ^***^ *p* < 0.001 vs. NC; ^##^ *p* < 0.01, ^###^ *p* < 0.001 vs. DKD1. NC, normal control; DKD, diabetic kidney disease; IHC, immunohistochemistry; IF, immunofluorescence.

A total of 17, 155, 27 genes were identified in the dataset. Principal component analysis (PCA) indicated that three groups of samples together were clustering (Supplementary Figure S1). A total of 31 genes related to necroptosis were found in AmiGO 2 (Supplementary Table S1). *Ripk 1*, *ripk 3*, and *mlkl* mRNA, as the key factors in the process of cell necroptosis, showed statistically significant changes and were especially increased in the DKD2 group (Figure 1A).

#### 3.1.2 Different necroptosis-related proteins are seen in human kidney tissue with diabetic kidney disease

The final calculated sample size was n = 3/group, based on the pre-experimental results of IHC staining for p-MLKL staining on glomeruli, the sample ratio (1:1:1), and the dropout rate (20%). The patients’ characteristics, which are described in Table 2, revealed no significant differences in age and gender among groups, while SBP and DBP in DKD2 group were higher than in NC and DKD1 group.

**TABLE 2:** The general characters of all patients.

Parameters	NC(*n* = 6)	DKD		*p*-value
		DKD1 (*n* = 10)	DKD2 (*n* = 13)	
Age (y)	51.50 (48.25, 54.75)	50.50 (32.75, 52.75)	48.00 (37.00, 53.00)	0.457
Gender (M/F)	4/2	6/4	10/3	0.688
SBP (mmHg)	117.50 ± 5.05	128.30 ± 30.53	149.69 ± 21.79^***^	0.001
DBP (mmHg)	70.00 ± 5.48	81.00 ± 10.80	93.31 ± 14.10^**#^	0.001
U-ACR (mg/gcr)	18.96 ± 5.75	85.06 ± 72.75^&^	3,324.77 ± 2031.77^***^	0.000
p-MLKL	7.44 ± 0.21	9.85 ± 0.88	26.44 ± 2.24***^###^	0.000

NC, vs. DKD1 ^&^ *p*< 0.05, NC, vs. DKD2^**^ *p*< 0.01,^***^ *p*< 0.001; DKD1 vs. DKD2 ^#^ *p*< 0.05,^###^ *p*< 0.001; M, male; F, female; SBP, systolic blood pressure; DBP, diastolic blood pressure; U-ACR, urine albumin creatinine ratio; p-MLKL, phosphorylated mixed-lineage kinase domain-like protein.

The expressions of p-MLKL protein were used as a marker for observing the changes of necroptosis in Human kidney biopsy tissue samples. Because albuminuria in DKD is mainly associated with glomerular damage, this study mainly measured necroptosis-related proteins in the glomeruli. The results of IHC staining indicated that p-MLKL protein was expressed in glomeruli and part of renal tubules, especially in the DKD2 group (Figure 1B and Table 2). Interestingly, the results of double immunofluorescence staining indicated that SYNPO, as a podocyte marker, partially overlapped with p-MLKL protein expression (Figure 1C).

#### 3.1.3 Preliminary exploration of the clinical significance of programmed necrosis in diabetic kidney disease development

In this part of the study, we first briefly compared the clinicopathological data of the two groups of patients in the DKD group. The results indicated significant differences in CHO, LDL, ALB, ApoB, glomerular lesions, IFTA, and interstitial inflammation compared to DKD1 with DKD2 groups (Table 3). The results of correlation analysis between p-MLKL protein and clinicopathological difference indicators indicated that p-MLKL was positively correlated with SBP, CHO, LDL, ApoB, and IFTA, while negatively correlated with ALB (Table 4).

**TABLE 3:** The clinical and pathological characters of DKD patients.

Parameters	DKD1 (*n* = 10)	DKD2 (*n* = 13)	t (Z)	*p*-value
DM duration (M)	74.60 ± 64.19	107.69 ± 74.73	1.117	0.276
Scr (μmol/L)	102.95 (49.55, 131.13)	118.40 (93.40,150.40)	(0.806)	0.420
BUN (mmol/L)	8.12 ± 2.01	8.83 ± 3.48	0.575	0.572
UA (μmol/L)	306.10 ± 76.38	352.92 ± 101.82	1.213	0.239
eGFR [ml/min·1.73 m^2^]	85.30 ± 36.15	70.77 ± 33.90	0.990	0.333
FBS (mmol/L)	5.54 (4.03, 6.25)	6.84 (5.34, 8.90)	(1.210)	0.226
HbAc1 (%)	7.10 (6.58, 7.70)	7.60 (6.10.8.00)	(0.279)	0.780
CHO (mmol/L)	4.31 ± 1.32	5.92 ± 1.08	3.221	0.004
TG (mmol/L)	1.95 (1.20.2.27)	1.50 (1.37.2.30)	(0.620)	0.535
LDL (mmol/L)	2.46 ± 1.27	3.88 ± 0.85	3.201	0.004
VLDL (mmol/L)	0.72 (0.45.0.84)	0.56 (0.51.0.85)	(0.559)	0.576
HDL (mmol/L)	1.03 ± 0.36	1.24 ± 0.27	1.616	0.121
ALB (g/L)	41.66 ± 2.30	30.68 ± 6.86	-5.393	0.000
ApoA1 (mmol/L)	1.14 (1.01.1.22)	1.30 (1.15.1.40)	(1.850)	0.064
ApoB (mmol/L)	0.86 ± 0.27	1.18 ± 0.28	2.702	0.014
ApoB/ApoA1	0.77 ± 0.24	0.95 ± 0.30	1.440	0.166
Lipoprotein α(mmol/L)	111.00 (46.00,253.00)	328.50 (166.25,626.75)	(1.845)	0.065
Glomerular lesions [n (%)]			(3.052)	0.002
Ⅰ	0 (0)	0 (0)		
Ⅱ				
Ⅱa	6 (60.00%)	1 (7.69%)		
Ⅱb	3 (30.00%)	3 (23.08%)		
Ⅲ	1 (10.00%)	9 (69.23%)		
Ⅳ	0	0		
IFTA (0/1/2/3)	3/4/3/0	0/2/9/2	(2.810)	0.005
Interstitial inflammation (0/1/2)	4/6/0	1/7/5	(2.276)	0.023
Arteriolar hyalinosis (0/1/2)	1/6/3	1/4/8	(1.344)	0.179
Arteriosclerosis (0/1/2)	0/8/2	1/7/5	(0.595)	0.552

DM, diabetic metabolism; M, month; NC, normal control; Scr, serum creatinine; BUN, blood urea nitrogen; UA, uric acid; eGFR, estimate glomerular filtration rate; FBS, fasting blood sugar; CHO, cholesterol; TG, triglycerides; LDL, low-density lipoprotein; VLDL, very-low-density lipoprotein; HDL, high-density lipoprotein; ALB, albumin; ApoB, apolipoprotein B; ApoA1, apolipoprotein A1; IFTA, interstitial fibrosis and tubular atrophy.

**TABLE 4:** Correlation between p-MLKL in renal tissue and clinical differential indicators.

Parameter	Correlation coefficient (r)	*P*
SBP	0.453	0.031
DBP	0.362	0.362
CHO	0.475	0.022
LDL	0.437	0.038
ALB	−0.749	0.000
ApoB	0.472	0.031
Glomerular lesions	0.408	0.054
IFTA	0.483	0.020
Interstitial inflammation	0.358	0.094

SBP, systolic blood pressure; DBP, diastolic blood pressure; CHO, cholesterol; LDL, low-density lipoprotein; ALB, albumin; ApoB, apolipoprotein B; IFTA, interstitial fibrosis and tubular atrophy.

### 3.2 Paeoniflorin improves kidney injury caused by diabetic kidney disease *in vitro* and *in vivo*

#### 3.2.1 Paeoniflorin shows renoprotection in streptozotocin-induced diabetic mice

**FIGURE 2:**
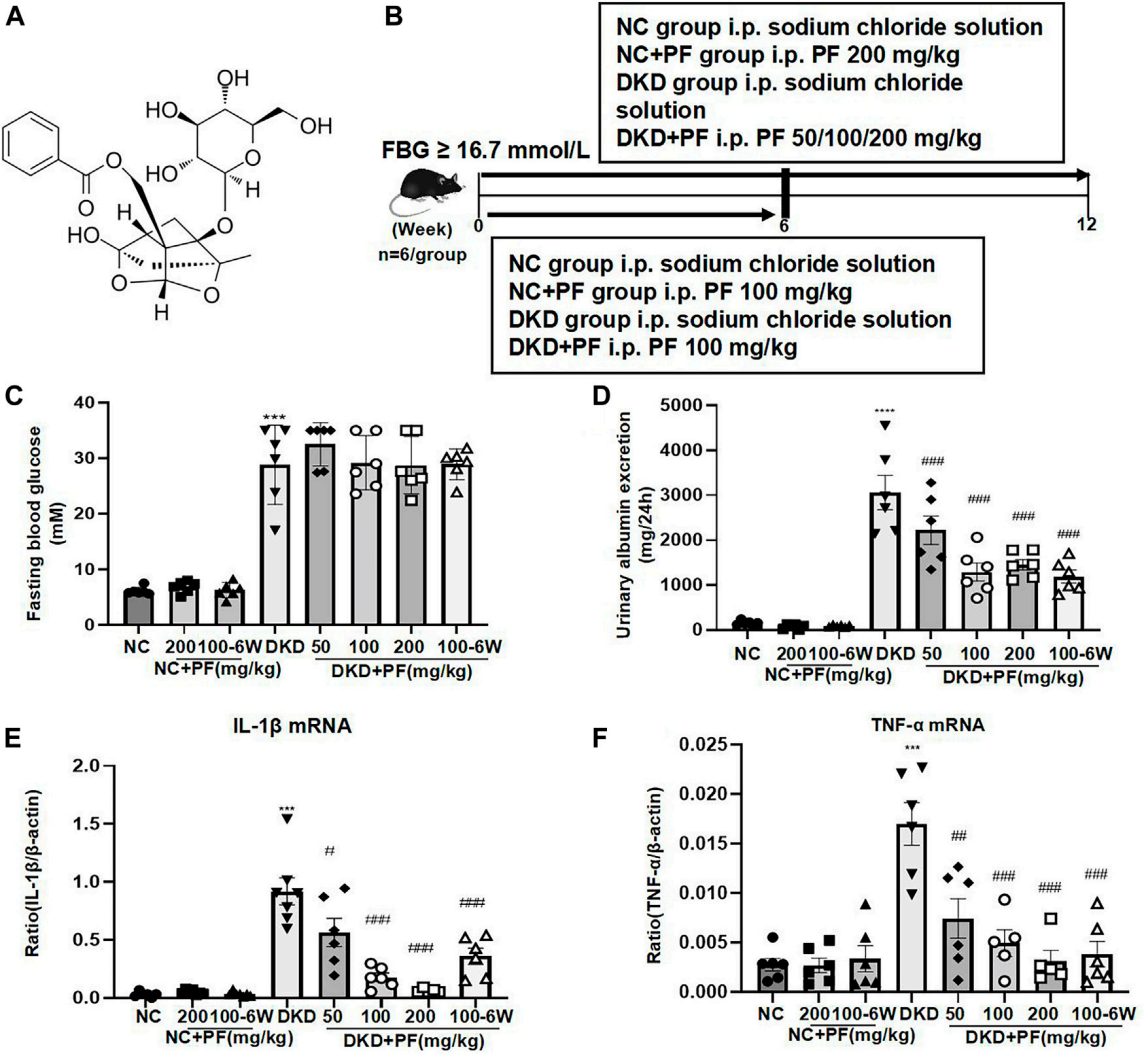
Serum and urine biochemical parameters of STZ-induced diabetic mice. **(A)** Molecular structure of PF. **(B)** Study design of the animal experiment and treatment. **(C)** Fasting blood glucose in different groups. **(D)** A 24 h urine albumin excretion in different groups. **(E)** IL-1β mRNA in different groups. **(F)** TNF-α mRNA in different groups. Results represent the mean ± SD for 6 mouse/group. ^***^ *p* < 0.001 vs. NC; ^#^ *p* < 0.05, ^##^ *p* < 0.01, ^###^ *p* < 0.001 vs. DKD. FBG, fasting blood glucose; NC, normal control; DKD, diabetic kidney disease; PF, paeoniflorin; STZ, streptozotocin; TNF-α, tumor necrosis factor-α.

The molecular structure of PF is shown in Figure 2A. We first observed the renoprotective effect of PF in mouse models of DKD (Figure 2B). The results of physiological and biochemical indicators showed that compared with the NC group, DKD group exhibited significantly higher levels of FBG, 24 UAER, and inflammatory markers of renal tissue, including TNF-a and IL-1β mRNA. Compared to the DKD group, 24 UAER and inflammatory markers of renal tissue were all decreased in a dose-dependent manner in PF groups (Figures 2C–F). There was no significant difference in Scr, BUN, ALB, ALT, and kidney/body ratio among groups (Supplementary Figures 2A–E).

**FIGURE 3:**
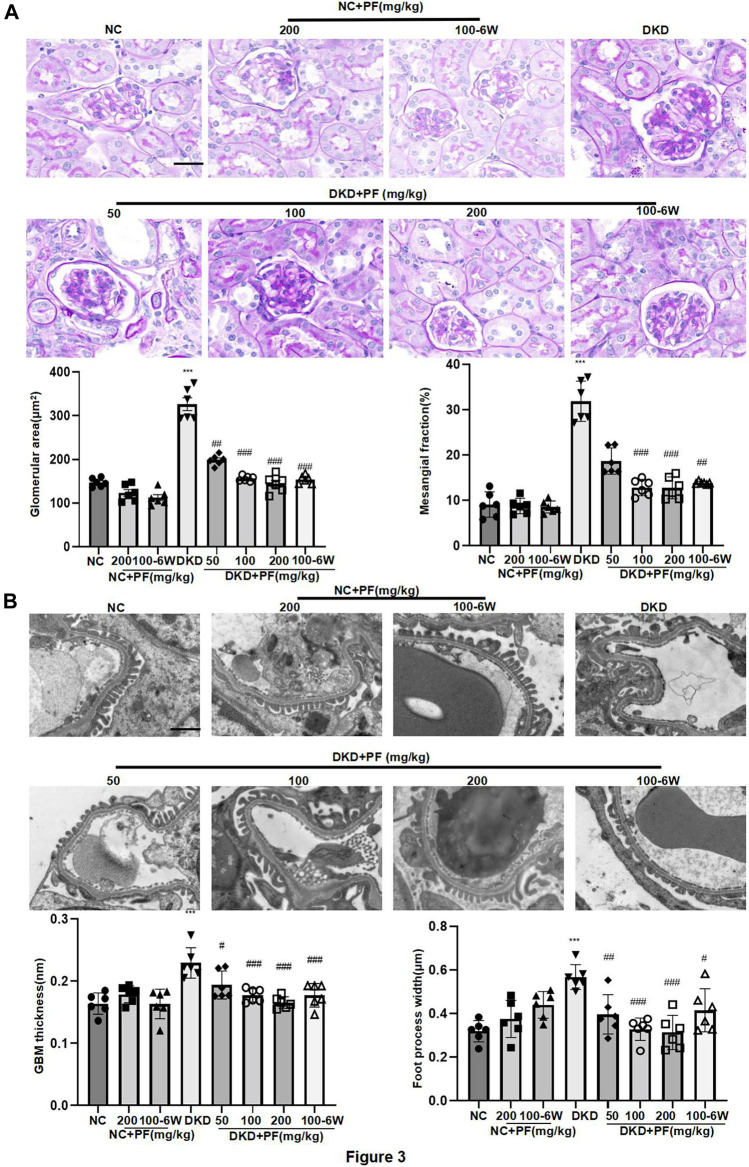
Morphological changes of STZ-induced diabetic mice. **(A)** Histopathological examinations of renal tissue sections stained with PAS from different groups, Scale bar = 50 μm. **(B)** Images of podocyte foot processes and glomerular basement membrane observed by TEM, Scale bar = 1 μm. Results represent the mean ± SD for 6 mouse/group. ^*^ *p* < 0.05, ^***^ *p* < 0.001 vs. NC; ^##^ *p* < 0.01, ^###^ *p* < 0.001 vs. DKD. NC, normal control; PF, paeoniflorin; STZ, streptozotocin; PAS, periodic acid–Schiff; TEM, transmission electron microscopy.

Observation of pathological kidney morphology further showed that compared with NC group, the glomerular volume enlarged, the mesangial area widened, foot process width increased and glomerular basement membrane (GBM) thickened in STZ-induced mouse DKD model, those were significantly alleviated after PF treatment (Figure 3). Interestingly, PF improved biochemical and pathological changes in DKD mice in a dose-dependent manner. In STZ-induced diabetic mouse model, PF showed renal protection after only 6 weeks of administration.

#### 3.2.2 PF ameliorates podocyte injury in diabetic kidney disease

##### 3.2.2.1 Paeoniflorin ameliorates podocyte injury in streptozotocin-induced diabetic mice

**FIGURE 4:**
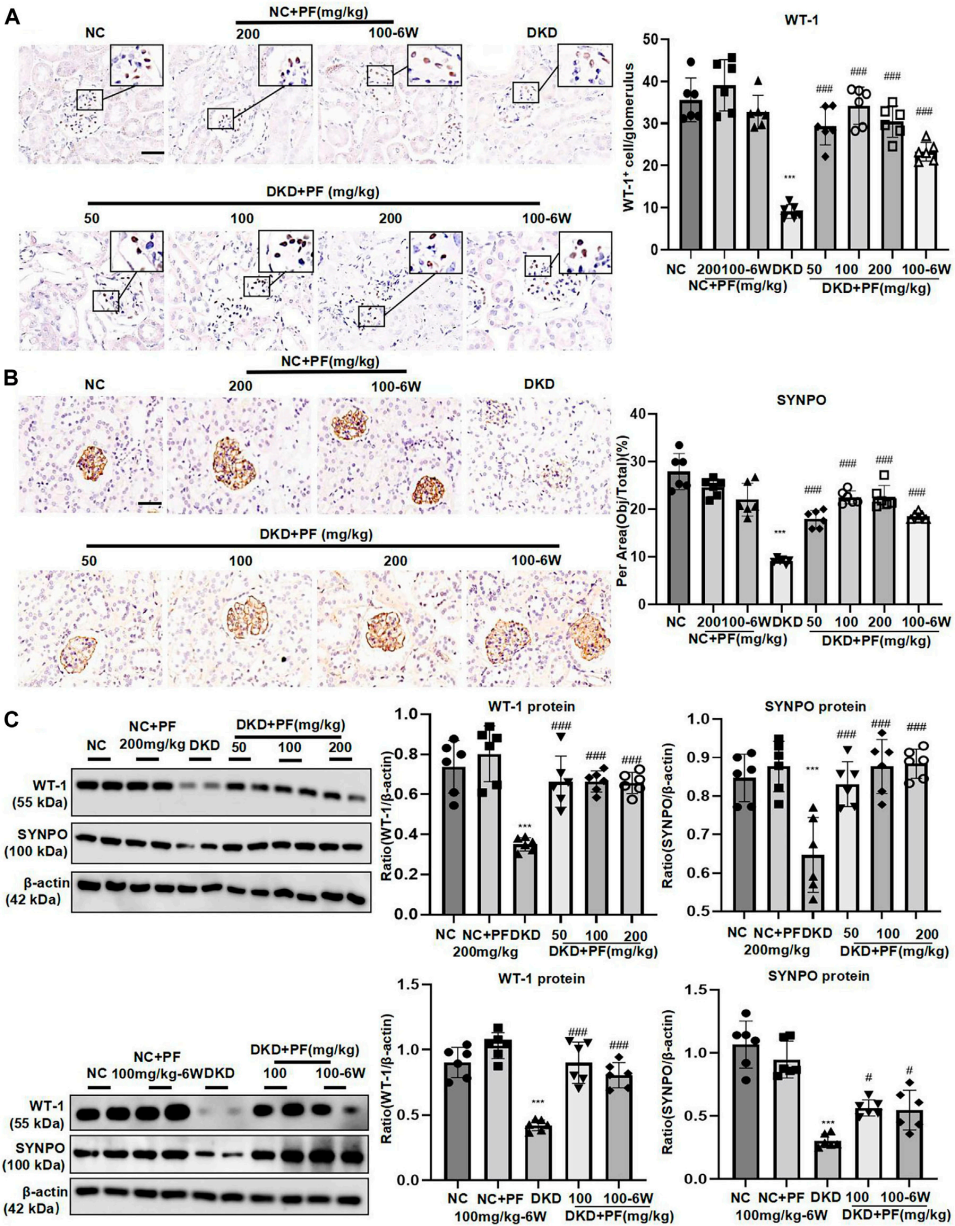
PF ameliorates podocyte injury in STZ-induced diabetic mice. **(A)** IHC assay of WT-1 protein expression in the glomerulus. Scale bar = 50 μm. **(B)** IHC assay of SYNPO protein expression in the glomerulus. Scale bar = 50 μm. **(C)** WB assay of WT-1 and SYNPO protein expression in the glomerulus. Data represent the mean ± SD for 6 mouse/group. ^***^ *p* < 0.001 vs. NC; ^#^ *p* < 0.05, ^###^ *p* < 0.001 vs. DKD. NC, normal control; PF, paeoniflorin; STZ, streptozotocin; DKD, diabetic kidney disease; WT-1, Wilms tumor 1 protein; SYNPO, synaptopodin; IHC, immunohistochemistry; WB, Western blotting.

We examined the protective effect of PF against podocyte injury, a key factor in the pathogenesis of albuminuria during DKD. The results of TEM detection showed that PF could significantly restore the fused foot processes of DKD mice (Figure 3B). Furthermore, the podocyte marker (WT-1) and the podocyte functional protein (SYNPO) were observed by IHC staining and WB. The results showed that the number and function of podocytes were significantly decreased in the STZ-induced mice model, but not in mice treated with PF (Figures 4A–C).

##### 3.2.2.2 Paeoniflorin ameliorates high glucose-stimulated podocyte injury

**FIGURE 5:**
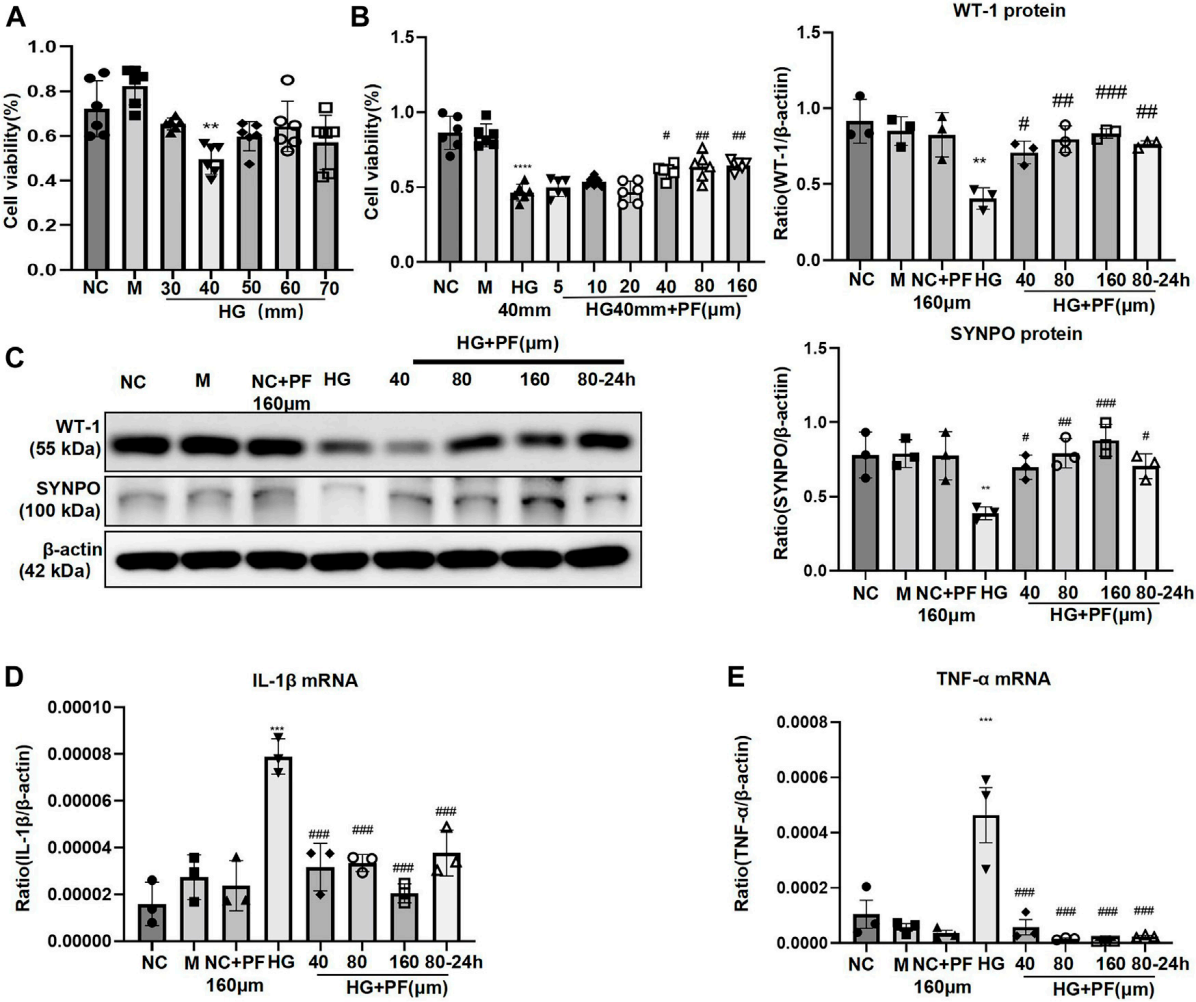
PF ameliorates HG-stimulated podocyte injury. **(A)** MTT assay to evaluate the effect of HG on MPC5 cells viability. **(B)** MTT assay to evaluate the effect of PF on HG-stimulated MPC5 cells viability. **(C)** WB assay of WT-1 and SYNPO protein in MPC5 cells. **(D)** IL-1β mRNA in MPC5 cells. **(E)** TNF-α mRNA in MPC5 cells. Results represent the mean ± SD. ^**^ *p* < 0.01, ^***^ *p* < 0.001 vs. NC; ^#^ *p* < 0.05, ^##^ *p* < 0.01, ^###^ *p* < 0.001 vs. HG. NC, normal control (5.5 mmol/L glucose); HG, high glucose (40 mmol/L); M, mannitol (34.5 mmol/L mannitol +5.5 mmol/L glucose); PF, paeoniflorin; WT-1, Wilms tumor 1 protein; SYNPO, synaptopodin; WB, Western blotting; MPC5, mouse podocyte clone 5; TNF-α, tumor necrosis factor-α.

MTT experiments indicated that 40 mmol/L was the optimal glucose concentration for high glucose-induced podocyte injury (Figure 5A), and 40 μmol/L is the minimum concentration of PF for improving podocyte proliferation (Figure 5B). *In vitro*, the experiments further showed that the expression of WT-1 and SYNPO proteins significantly decreased on podocytes induced by HG (Figure 5C), and the mRNAs of inflammatory factors TNF and IL-1β were significantly increased (Figures 5D,E). Contrary, the above changes in podocytes obviously improved after PF treatment in a dose-dependent manner. In addition, the middle dose (80 μmol/L) was further selected to observe the preventive effect of PF on HG-induced podocyte injury. Similar to the animal experiments, podocyte injury and inflammatory responses were alleviated after PF preventive treatment.

### 3.3 Paeoniflorin regulates podocyte necroptosis of diabetic kidney disease *in vitro* and *in vivo*

#### 3.3.1 Paeoniflorin regulates podocyte necroptosis in streptozotocin-induced diabetic mice

**FIGURE 6:**
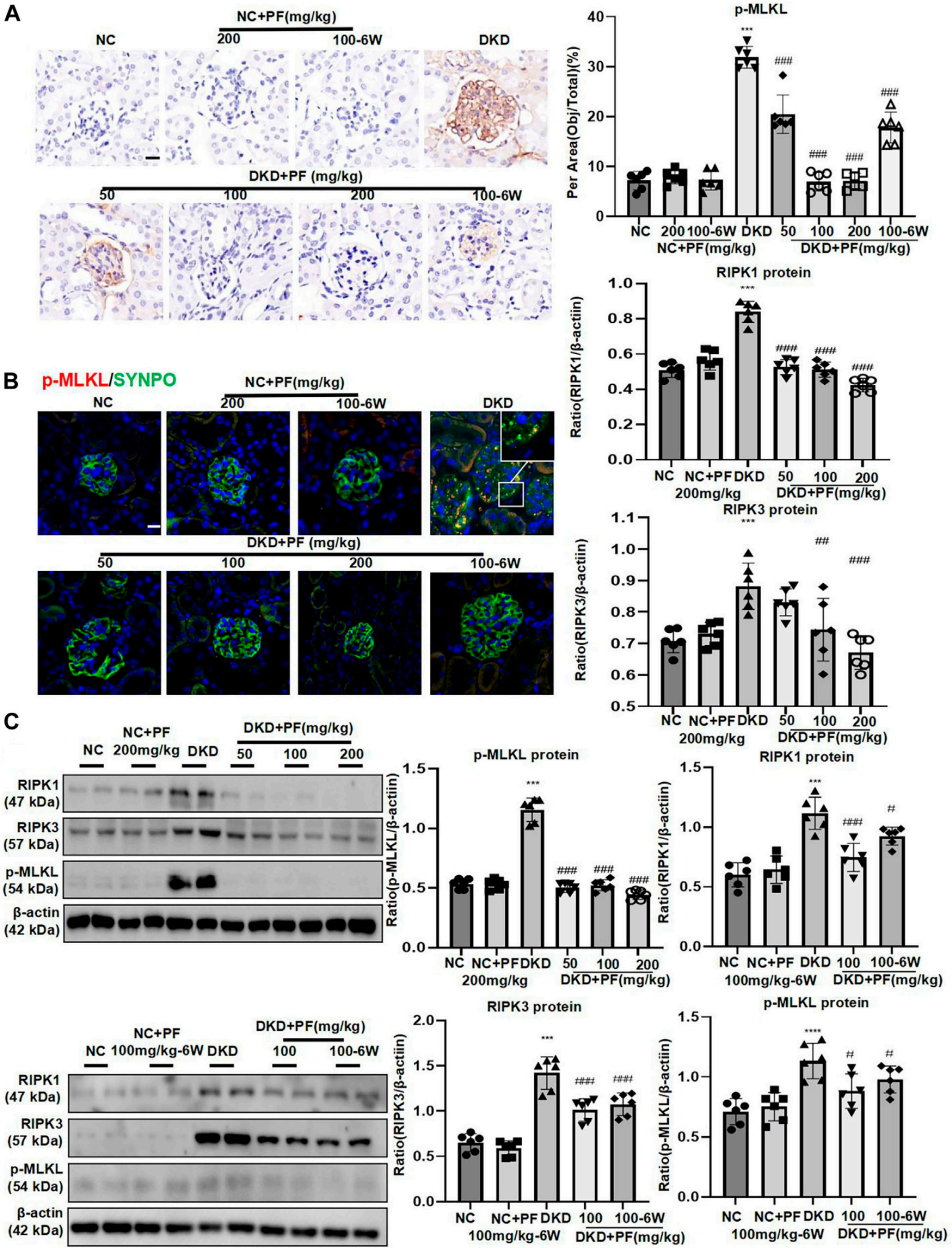
PF regulates podocyte necroptosis in STZ-induced diabetic mice. **(A)** IHC assay of p-MLKL protein expression on the glomerulus in STZ-induced diabetic mice. Scale bar = 50 μm. **(B)** IF double staining of p-MLKL and SYNPO proteins expression in renal biopsy samples. Scale bar = 50 μm. **(C)** WB assay of RIPK1, RIPK3, and p-MLKL proteins in STZ-induced diabetic mice. Data represent the mean ± SD for 6 mouse/group. ^***^ *p* < 0.001 vs. NC; ^#^ *p* < 0.05, ^##^ *p* < 0.01, ^###^ *p* < 0.001 vs. DKD. NC, normal control; PF, paeoniflorin; STZ, streptozotocin; DKD, diabetic kidney disease; IHC, immunohistochemistry; IF, immunofluorescence; WB, Western blotting; SYNPO, synaptopodin; RIPK1, receptor-interacting serine/threonine kinase 1; RIPK3, receptor-interacting serine/threonine kinase 3; MLKL, mixed-lineage kinase domain-like protein.

In STZ-induced diabetic mice models, the p-MLKL protein was mainly expressed on glomeruli and was obviously higher than that in the NC group, which was consistent with clinical data. Contrary, p-MLKL was downregulated especially in the middle and high dose groups of PF. There was no significant difference between NC group and PF group. Research also indicated that IHC results of p-MLKL protein were consistent with WB detection results (Figures 6A,C). IF double staining results showed that SYNPO was co-expressed with p-MLKL in the DKD mice model group, suggesting that necroptosis occurred on podocytes during DKD development (Figure 6B). WB also detected the marker proteins RIPK1 and RIPK3 of the programmed necrosis signaling pathway, suggesting that the protein expression was dramatically increased in DKD group, while decreased after the treatment of PF in a dose-dependent manner. The levels of necroptosis-related proteins also showed similar changes in the PF preventive treatment group with the full course of treatment (Figure 6C).

#### 3.3.2 Paeoniflorin regulates podocyte necroptosis stimulated by high glucose

**FIGURE 7:**
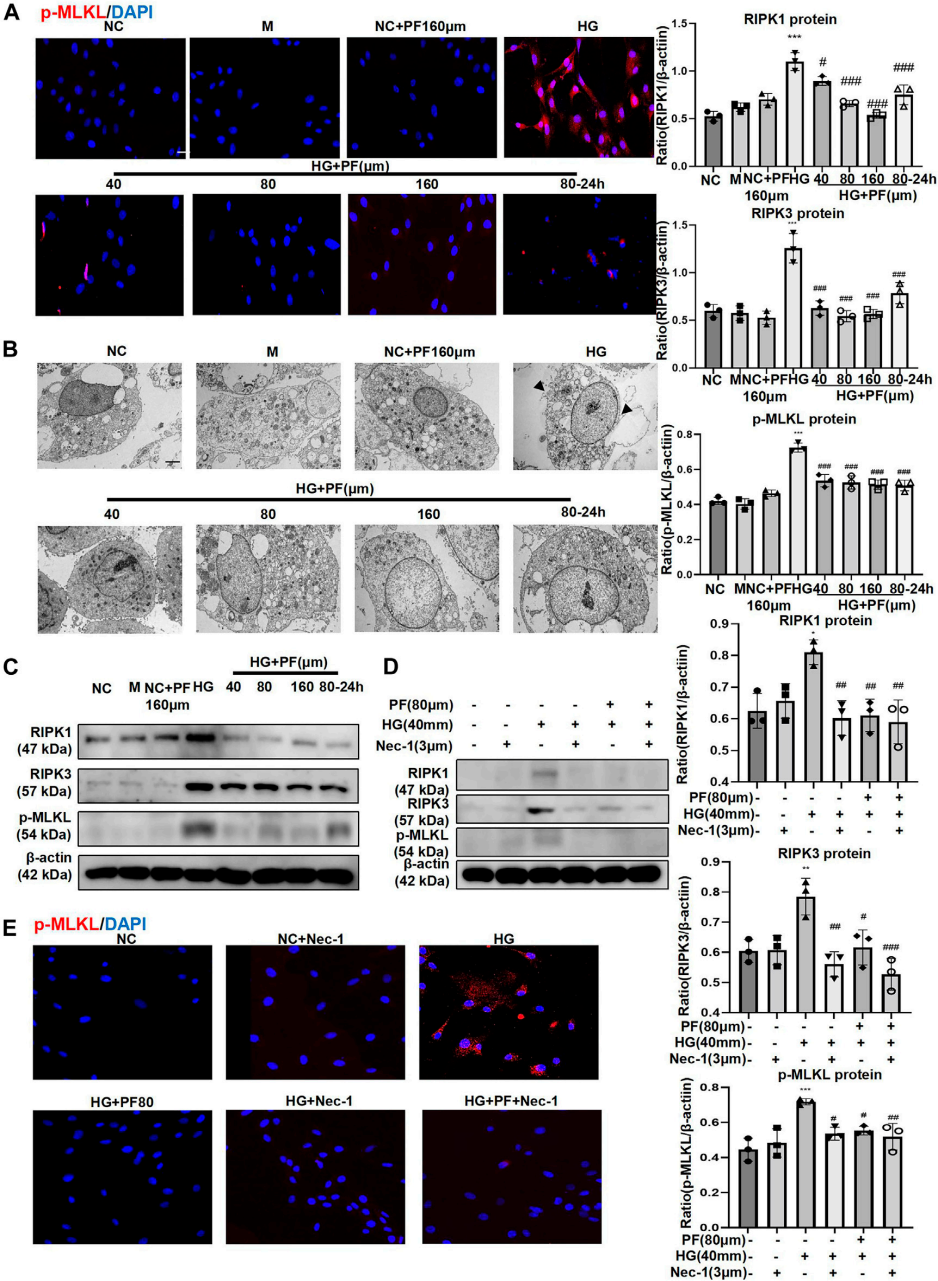
PF regulates podocyte necroptosis stimulated by HG. **(A)** IF staining of p-MLKL proteins expression in MPC5 cells stimulated with HG and treated with PF. Scale bar = 50 μm. **(B)** Images of MPC5 cells observed by TEM. **(C)** WB assay of RIPK1, RIPK3, and p-MLKL proteins in MPC5 treated with HG and PF. **(D)** WB assay of RIPK1, RIPK3, and p-MLKL proteins in MPC5 treated by Nec-1. **(E)** IF staining of p-MLKL proteins expression in MPC5 treated by Nec-1. Scale bar = 50 μm. Results represent the mean ± SD. ^*^ *p* < 0.05, ^**^ *p* < 0.01, ^***^ *p* < 0.001 vs. NC; ^#^ *p* < 0.05, ^##^ *p* < 0.01, ^###^ *p* < 0.001 vs. HG. NC, normal control (5.5 mmol/L glucose); HG, high glucose (40 mmol/L); M, mannitol (34.5 mmol/L mannitol +5.5 mmol/L glucose); PF, paeoniflorin; WB, Western blotting; MPC5, mouse podocyte clone 5; Nec-1, necrostatin-1; TEM, transmission electron microscopy; RIPK1, receptor-interacting serine/threonine kinase 1; RIPK3, receptor-interacting serine/threonine kinase three; MLKL, mixed-lineage kinase domain-like protein.

We further observed the effect of PF on the necroptosis of podocytes induced by HG. IF staining showed that p-MLKL protein level in podocytes significantly increased when induced by HG while decreased after PF treatment (Figure 7A). Furthermore, the observation using TEM showed that the cell membrane integrity of podocytes was damaged by HG-stimulation group, but not in the PF group (Figure 7B).

Moreover, we further detected necroptosis signaling pathway-related proteins by the WB method; the results were consistent with the cell IF results (Figure 7C). It should be mentioned that the changes related to necroptosis in PF groups were dose-dependent, and prevention treatment of PF also exhibited necroptosis modulating effects. Additionally, Nec-1, as an inhibitor of necroptosis, was used as a positive control to confirm that the performance of PF in inhibiting necroptosis of podocytes was similar to that of Nec-1 (Figures 7D,E).

### 3.4 Paeoniflorin regulates TNFR1 protein expression of diabetic kidney disease *in vitro* and *in vivo*

#### 3.4.1 The changes of TNFR1 in diabetic kidney disease

**FIGURE 8:**
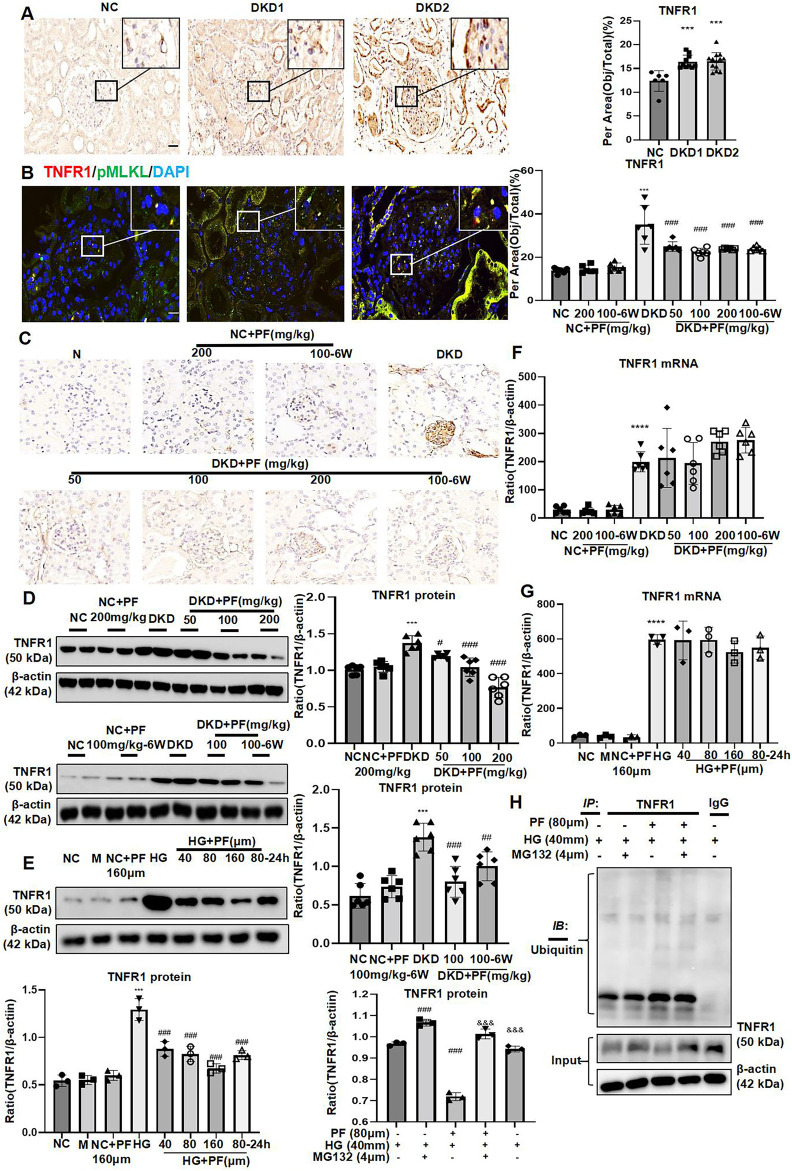
PF regulates TNFR1 protein expression *in vivo* and *in vitro*. **(A)** IHC assay of TNFR1 protein expression on the glomerulus of human renal biopsy tissues. Scale bar = 20 μm. **(B)** IF double staining for p-MLKL and TNFR1 in human renal biopsy tissues. Scale bar = 20 μm. **(C)** IHC assay of TNFR1 protein expression on the glomerulus in STZ-induced diabetic mice. Scale bar = 50 μm. **(D)** WB assay of TNFR1 protein in STZ-induced diabetic mice. **(E)** WB assay of TNFR1 protein in MPC5 with HG and PF treatment. **(F)** The levels of TNFR1 mRNA in STZ-induced diabetic mice. **(G)** The levels of TNFR1 mRNA in MPC5 with HG and PF treatment. **(H)** The ubiquitination of TNFR1 in MPC cells. Results represent the mean ± SD. ^**^ *p* < 0.01, ^***^ *p* < 0.001 vs. NC; ^#^ *p* < 0.05, ^##^ *p* < 0.01, ^###^ *p* < 0.001 vs. HG/DKD; ^&&&^ *p* < 0.001 vs. HG+PF. NC, normal control (5.5 mmol/L glucose); HG, high glucose (40 mmol/L); M, mannitol (34.5 mmol/L mannitol +5.5 mmol/L glucose); PF, paeoniflorin; STZ, streptozotocin; DKD, diabetic kidney disease; IHC, immunohistochemistry; TNFR1, tumor necrosis factor receptor 1; IF, immunofluorescence; WB, Western blotting; MPC5, mouse podocyte clone 5; RIPK1, receptor-interacting serine/threonine kinase 1; RIPK3, receptor-interacting serine/threonine kinase 3; MLKL, mixed-lineage kinase domain-like protein.

IHC staining showed that TNFR1 protein was expressed in glomeruli and renal tubules in human kidney tissue. Furthermore, in the NC group, there was a small amount of TNFR1 expression on the glomerulus, which gradually increased with increased urinary albumin (Figure 8A). The results of IF double staining indicated that TNFR1 and p-MLKL co-localized in the glomerulus (Figure 8B).

#### 3.4.2 Paeoniflorin regulates TNFR1 protein expression in streptozotocin-induced diabetic mice and podocytes stimulated by high glucose

IHC and WB showed that the expression of TNFR1 was low under the normal condition, but was increased mainly in the glomerulus of STZ-induced diabetic mice. TNFR1 was dramatically decreased after PF treatment, similar to NC group (Figures 8C,D).

Similarly, TNFR1 protein expression in podocytes was significantly increased after HG-stimulation and decreased after PF treatment (Figure 8E). Interestingly, the experimental results found no change in the level of TNFR1 mRNA after PF treatment *in vitro* and *in vivo* (Figures 8F,G).

Moreover, this study detected the ubiquitination level of TNFR1, purified by COIP method, suggesting that the administration of PF significantly increased the ubiquitination level of TNFR1 protein induced by HG (Figure 8H).

### 3.5 Paeoniflorin binds directly to TNFR1

**FIGURE 9:**
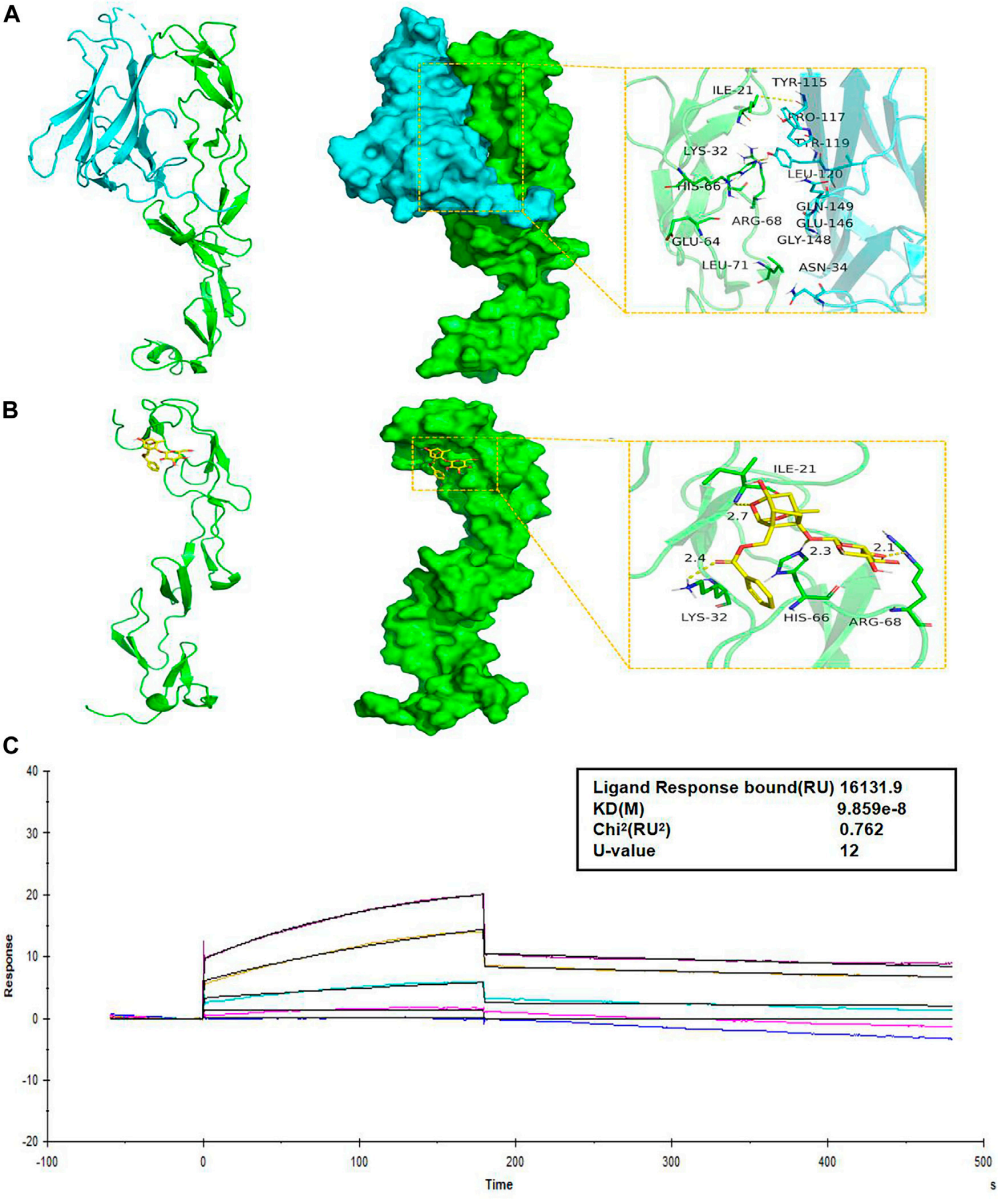
PF directly binds to TNFR1. **(A)** The binding mode of the complex TNFRSF1A with TNF. The backbone of protein was rendered in a tube and colored in green. TNFRSF1A (left) and TNF (right) protein were rendered by the surface. The yellow dash represents a hydrogen bond or salt bridge. **(B)** The binding mode of the complex TNFRSF1A with PF. The backbone of protein was rendered in a tube and colored in green. TNFRSF1A protein was rendered by the surface. The yellow dash represents a hydrogen bond or salt bridge. **(C)** SPR assay showed the steady-state fit of binding between PF and TNFR1. TNFR1, tumor necrosis factor receptor 1; TNFRSF1A, tumor necrosis factor receptor superfamily member 1; PF, paeoniflorin; SPR, surface plasmon resonance.

Molecular structure screening in Molecular docking is shown in Table 5. We first analyzed the binding mode of TNFR1 to its ligand TNF. The binding score of TNFRSF1A and TNF protein was -56.59 kcal/mol. The binding sites of TNFRSF1A protein included ILE-21, LYS-32, HIS-66, ARG-68, GLU-64, LEU-71, and other amino acid residues, while the binding sites of TNF included TYR-115, PRO-117, TYR-119, LEU-120, GLN-149, GLU-146, ASN-34, and other amino acid residues. The interaction of these reactive amino acids has an important role in stabilizing the two proteins (Figure 9A). Therefore, the amino acids that interact with the two proteins can be used as active sites to bind to small molecules, thereby hindering the binding of the two proteins.

**TABLE 5:** The molecular docking results of target proteins.

Protein1	Protein2	Binding energy (kcal/mol)	Contact sites (protein1)	Contact sites (protein2)	Combination type
TNFRSF1A	TNF	-56.59	ILE-21, LYS-32, HIS-66, ARG-68, GLU-64, LEU-71	TYR-115, PRO-117, TYR-119, LEU-120, GLN-149, GLU-146, ASN-34	Hydrogen bond, Hydrophobic interaction
TNFRSF1A	PF	-8.32	ILE-21, LYS-32, HIS-66, ARG-68	-	Hydrogen bond, Hydrophobic interaction

PF, paeoniflorin; TNFR1, tumor necrosis factor receptor 1; TNFRSF1A, tumor necrosis factor receptor superfamily, member 1.

Next, we analyzed the interaction of PF with TNFRSF1A. The main binding sites of PF to TNFRSF1A target were ILE-21, LYS-32, HIS-66, ARG-68, and other amino acid residues. The binding score of PF and TNFRSF1A protein was −8.32 kcal/mol. PF compound contains multiple hydrogen bond donors and acceptors, which can form strong hydrogen bond interactions with the active groups of amino acids such as ILE-21, LYS-32, HIS-66, ARG-68 and so on. The average hydrogen bond distance was 2.5 Å. In addition, the hydrophobic benzene ring of this compound could also form a strong hydrophobic interaction with the amino acid in the active pocket, which was very helpful for stabilizing small molecules (Figure 9B). Therefore, the PF compound had a high degree of matching with the active pocket of TNFRSF1A protein and could form multiple strong hydrogen bond interactions with amino acids in the active site, which had a role in hindering the binding of TNFRSF1A and TNF. In addition, the SPR assay demonstrated the direct binding between PF and TNFR1 protein, with an equilibrium dissociation constant (KD) of 9.859 × 10^−8^ for steady-state fit, and the combination or separation of PF and TNFR1 was relatively rapid (Figure 9C).

### 3.6 Paeoniflorin regulates podocyte necroptosis via TNFR1

**FIGURE 10:**
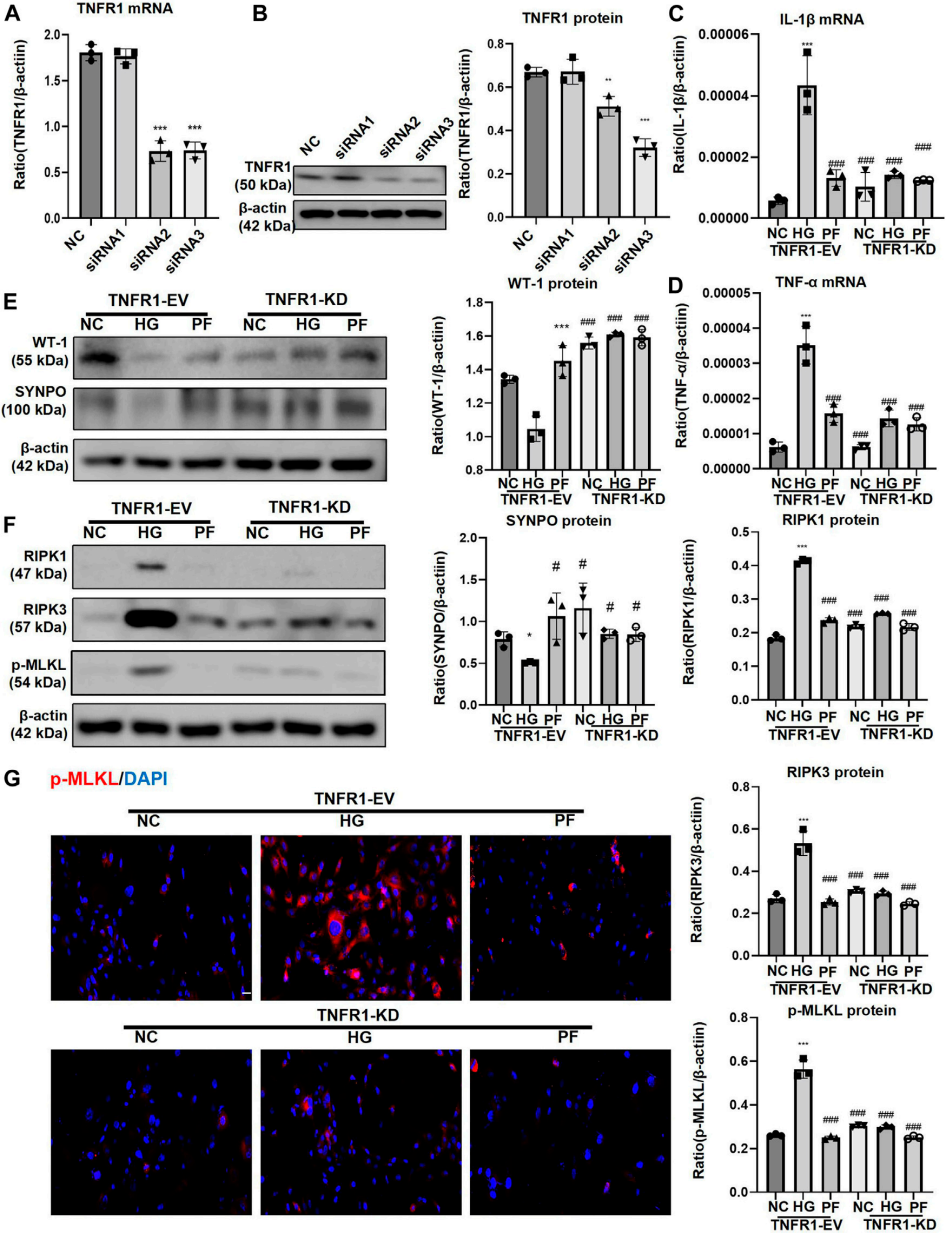
PF regulates podocyte necroptosis via TNFR1. **(A)** Real-time PCR assay of TNFR1 in MPC5 cells after TNFR1 knockdown. **(B)** WB assay of TNFR1 protein in MPC5 cells after TNFR1 knockdown. **(C)** The change of IL-1β mRNA in MPC5 cells after TNFR1 knockdown. **(D)** The change of TNF-α mRNA in MPC5 cells after TNFR1 knockdown. **(E)** WB assay of WT-1 and SYNPO proteins in MPC5 cells after TNFR1 knockdown. **(F)** WB assay of necroptosis-related proteins in MPC5 cells after TNFR1 knockdown. **(G)** IF staining of p-MLKL proteins expression in MPC5 cells after TNFR1 knockdown. Scale bar = 50 μm. Results represent the mean ± SD. ^*^ *p* < 0.05, ^***^ *p* < 0.001 vs. NC; ^#^ *p* < 0.05, ^##^ *p* < 0.01, ^###^ *p* < 0.001 vs. HG. NC, normal control (5.5 mmol/L glucose); HG, high glucose (40 mmol/L); PF, paeoniflorin; TNFR1, tumor necrosis factor receptor 1; IF, immunofluorescence; WB, Western blotting; MPC5, mouse podocyte clone 5; TNF-α, tumor necrosis factor-α; WT-1, Wilms tumor 1 protein; SYNPO, synaptopodin; RIPK1, receptor-interacting serine/threonine kinase 1; RIPK3, receptor-interacting serine/threonine kinase 3; MLKL, mixed-lineage kinase domain-like protein.

The effect of siRNAs on TNFR1 knockdown is shown in Figures 10A,B. After knockdown of TNFR1 on podocytes, the levels of inflammatory indicators (IL-1β and TNF-α mRNA), cell injury indicators (WT-1 and SYNPO protein), and necroptosis-related proteins were significantly reduced, similarly to the effect of PF on HG-stimulated podocytes, but the podocyte protection and necroptosis regulation of PF were not improved after TNFR1 knockdown (Figures 10C–F). The p-MLKL IF staining of the cell slides was consistent with the WB results (Figure 10G).

## 4 Discussion

The mechanism of occurrence and development of DKD is quite complex, requiring the participation of many cells and the regulation of various signaling pathways (Raval et al., 2020). Among them, the formation of proteinuria in the early stage of DKD is closely related to the damage of podocytes, which is irreversible (Sugita et al., 2021). Therefore, exploring podocyte death’s occurrence and development mechanism may provide a new way to treat and prevent DKD. To the best of our knowledge, this is the first study that reported the significance of necroptosis in the process of DKD and the inhibitory effect of PF on podocyte necroptosis in DKD through directly binding TNFR1. These results provided a theoretical basis for the application of PF in DKD.

Necroptosis is a form of the programmed cell death initiated by various pattern recognition receptors (PRRs) or cytokines, and mediated by RIPK1, RIPK3, and MLKL. Phosphorylated-MLKL (p-MLKL) acts as an executor that ultimately induces necroptosis by translocating and breaking the cell membrane and eventually resulting in cell death (Seo et al., 2019). In this study, we first observed the dynamic changes of necroptosis in the renal tissues of DKD patients, especially in the glomerulus. The RNA-seq dataset GSE142025 was selected to observe the dynamic changes of necroptosis-related genes in the process of DKD; RNA-seq dataset GSE142025 contains human renal biopsy samples with the microalbumin and macroalbuminuria stages of DKD. The mRNA levels of necroptosis-related genes were dramatically upregulated in DKD patients with macroalbuminuria. RIPK1, RIPK3, and MLKL were identified as key factors in developing necroptosis and the progression of DKD. Interestingly, from the analysis results of this dataset, the necroptosis did not seem to occur at the initial stage of DKD but at the time of progressive exacerbation. However, it should be noted that the actual occurrence of necroptosis requires the formation of p-MLKL, which will be used as a marker for more accurate evaluation of the level of necroptosis in our subsequent study. In addition, necroptosis observed by IHC staining of human kidney tissue was present not only in the tubulointerstitium as in acute kidney injury, but also in the glomerular resident cells of DKD patients, especially of those with macroalbuminuria. This result was consistent with the observations on the above datasets. Furthermore, IHC staining of renal tissue in an STZ-induced mouse diabetes model with macroalbuminuria was used to locate and observe necroptosis. The results showed that the change of p-MLKL was most significant in the glomerulus. This gave us an interesting hint that the glomerulus resident cells showed necroptosis markedly during macroalbuminuria. Therefore, this study focused on detecting necroptosis changes in the glomerulus. Results of correlation analysis showed that glomerulus necroptosis might participate in the progression of renal tissue damage in DKD.

In our study, the results of IF double staining confirmed the presence of necroptosis in podocytes in DKD. The *in vitro* experiments also showed classical changes associated with necroptosis, including the destruction of the cell membrane and elevated p-MLKL protein in podocytes stimulated by HG. Therefore, amelioration of necroptosis may be a potential measure to protect against podocyte damage in DKD.

In China, the clinical efficacy and safety of some classic Chinese herbal medicines have undergone long-term and sufficient clinical verification. Paeoniflorin has been studied for decades as the main active ingredient of the classic traditional Chinese medicine Paeonia lactiflora. Our previous study found that PF can improve kidney injury in DKD mice model without obvious toxic side effects (Zhang et al., 2017). Furthermore, we validated the advantages of PF for kidney and podocyte, finding that PF can improve renoprotection and alleviate podocyte injury in both the whole administration and prophylactic administration. All roads lead to Rome. These interesting results provided a certain basis for the mobility of PF in clinical application. In exploring the mechanism of PF in DKD, we found that PF can significantly inhibit glomerulus necroptosis in DKD, especially on podocytes, similarly to the classical necroptosis inhibitor, necrostatin-1 (Nec-1).

Subsequent studies focused on the specific mechanisms through which PF affects podocyte necroptosis in DKD. TNFRSF1A (also named TNFR1) was the interacting protein of RIPK1 predicted by the sting website (https://cn.string-db.org/). TNFRs are recognized as the receptor of TNFα, leading to the nitric oxide production (Jha et al., 2020). The levels of TNFR1 in circulation have been associated with the damage of kidney in type 2 diabetes (Fernandez-Real et al., 2012). Interestingly enough, we found in our study that the expression of TNFR1 protein on the glomerulus was obviously upregulated in human and mouse with DKD. Meanwhile, the trend of TNFR1 expression was consistent with the necroptosis marker, p-MLKL. Previous reports have pointed out that the intracellular domain of TNFR1 on the cell membrane can bind to RIPK1 and regulate the phosphorylation of MLKL through RIPK1/RIPK3 signaling pathway, thereby participating in cell necroptosis (Dondelinger et al., 2016). Thus, TNFR1 may be an upstream target protein of necroptosis on podocytes. Unfortunately, there is no report on the clinical application of TNFR1-specific blockers or inhibitors (Al-Lamki and Mayadas, 2015). Animal experiments showed that PF could downregulate the expression of TNFR1 protein on the glomerulus, which was consistent with *in vitro* data on podocytes. Molecular docking further showed that PF and TNFR1 had multiple binding sites, some of which overlapped with TNF-a and TNFR1 binding sites, suggesting that PF may compete with TNF-a to bind TNFR1 for regulating necroptosis. SPR experiments verified the rapid association and dissociation properties of PF with TNFR1, providing a certain basis for the safety of multiple administration of PF. However, it should be noted that in another report, CD40, which was upregulated in podocytes, tubular epithelial cells and various infiltrating cells of human renal tissue with DKD, can change TRAF-binding domains to affect the function of TNFRs. This result was mainly achieved through the effect of CD40^−^CD40L interacting residues on the binding capacity of TRAF (Sarode et al., 2020). Therefore, we wondered whether PF could also act in a similar manner as CD40.

Next, we explored the specific mechanism of PF in regulating TNFR1. Common mechanisms that affect protein expression include transcription and translation of protein-related genes and protein folding and degradation. The ubiquitin-proteasome system is the main pathway for intracellular protein degradation. The ubiquitination system, consisting of E1 (ubiquitin activation), E2 (ubiquitin binding/carrier), and E3 (ubiquitin ligase), can anchors proteins and transport them to the 26S proteasome for degradation (Wertz and Dixit, 2008). In this study, there was no significant change in TNFR1 mRNA level after PF treatment in DKD modes, suggesting that PF might directly affect the protein level of TNFR1. We tried to clarify the downregulation mechanism of PF in the expression of TNFR1 by focusing on the protein ubiquitination level leading to protein degradation. The further research suggested that the ubiquitination of TNFR1 protein increased in the PF group, suggesting that PF promotes the degradation of TNFR1 protein. Finally, after TNFR1 knockdown, the injury and necroptosis of cultured podocytes stimulated by HG decreased significantly, while PF could no longer exert its protective effects on podocyte injury and regulation of necroptosis. Therefore, we confirmed that PF could directly bind to TNFR1 and increase its degradation to regulate podocyte necroptosis mediated by the RIPK1/RIPK3 signaling pathway in DKD.

Admittedly, this study has a lot of limitations. First, the clinical sample size was not large enough. Also, observation of necroptosis was limited to renal tissue specimens; other types of specimens, including blood and urine, need to be examined in future studies. In addition, due to the limitation of intraperitoneal administration of PF, this study only used STZ-induced mice as a diabetic model. Further studies should be performed to evaluate the role of necroptosis in other types of DKD model.

## 5 Conclusion

PF can improve renoprotection and alleviate podocyte injury in both the whole administration and prophylactic administration. Furthermore, PF can directly bind and promote the degradation of TNFR1 in podocytes and then regulate the RIPK1/RIPK3 signaling pathway to affect necroptosis, thus preventing podocyte injury in DKD. Thus, TNFR1 may be used as a new potential target to treat DKD.

## Data Availability

The datasets presented in this study can be found in online repositories. The names of the repository/repositories and accession number(s) can be found in the article/Supplementary Material.

## Glossary

ACR: albumin to creatinine ratio
ALB: albumin;
ALT: serum alanine transaminase
ApoA1: apolipoprotein A1
ApoB: apolipoprotein B
BUN: blood urea nitrogen
CHO: cholesterol
CO-IP: co-immunoprecipitation assay
DBP: diastolic blood pressure
DEGs: differential expression genes
DKD: diabetic kidney disease
DM: diabetic metabolism
eGFR: estimate glomerular filtration rate
endMT: endothelial-mesenchymal transition
EMT: epithelial-mesenchymal transition
FBS: fasting blood sugar
FC: fold change
GBM: glomerular basement membrane
GFB: glomerular filtration barrier
HG: high glucose
HDL: high-density lipoprotein
IHC: immunohistochemistry
IF: immunofluorescence
IFTA: interstitial fibrosis and tubular atrophy
LDL: low-density lipoprotein
M: mannitol
MLKL: mixed-lineage kinase domain-like protein
MPC5: mouse podocyte clone 5
NC: normal control
Nec-1: necrostatin-1
OD: optical density
PF: paeoniflorin
RIPK1: receptor-interacting serine/threonine kinase 1
RIPK3: receptor-interacting serine/threonine kinase 3
SBP: systolic blood pressure
Scr: serum creatinine
SPR: surface plasmon resonance
SPF: specific pathogen-free
STZ: streptozotocin
SYNPO: synaptopodin
TEM: transmission electron microscopy
TNF-α: tumor necrosis factor-α
TNFR1: tumor necrosis factor receptor 1
TNFRSF1A: tumor necrosis factor receptor superfamily, member 1
TG: triglycerides
UAER: urinary albumin excretion rate
UA: uric acid
VLDL: very low-density lipoprotein
WB: western blotting
WT: wild-type
WT-1: wilms tumor 1 protein.
