# Supplementary figures and images for: Paeoniflorin directly binds to TNFR1 to regulate podocyte necroptosis in diabetic kidney disease
Source: Front Pharmacol. 2022 Sep 6;13:966645. doi: 10.3389/fphar.2022.966645 (PMC9486100; doi:10.3389/fphar.2022.966645)

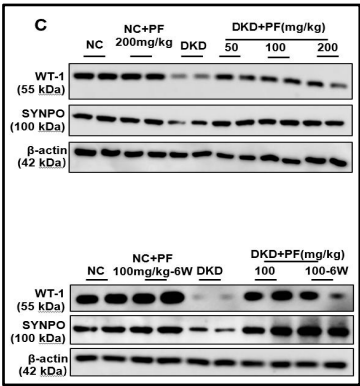

1

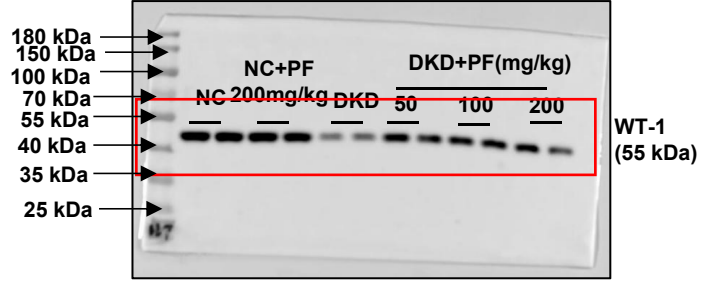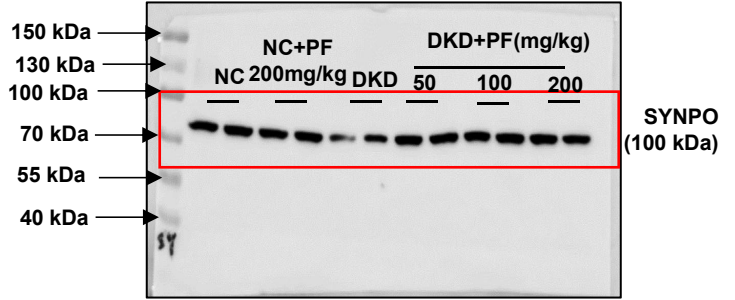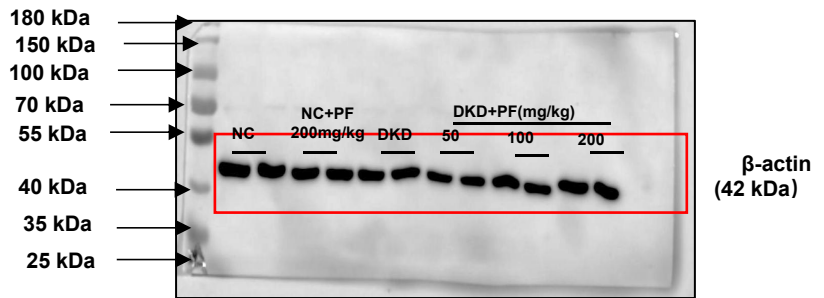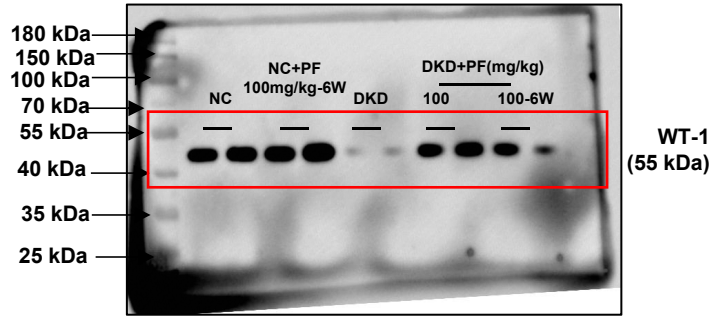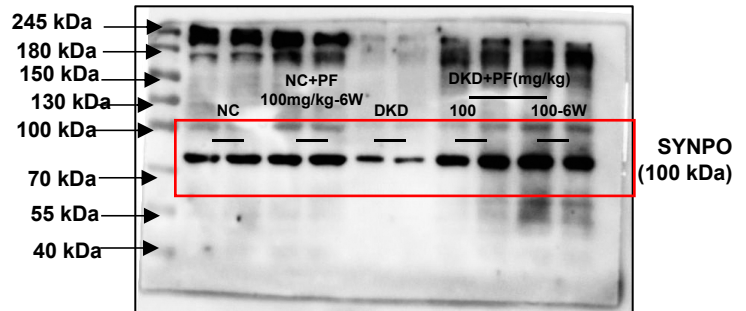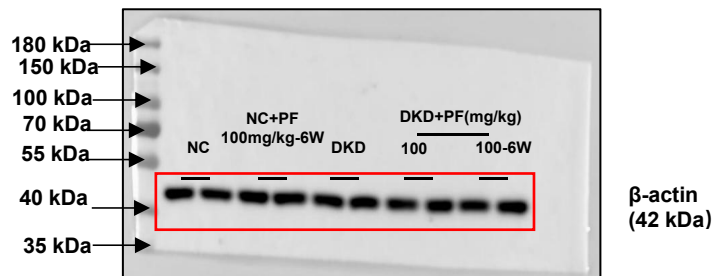

Figure 4 C

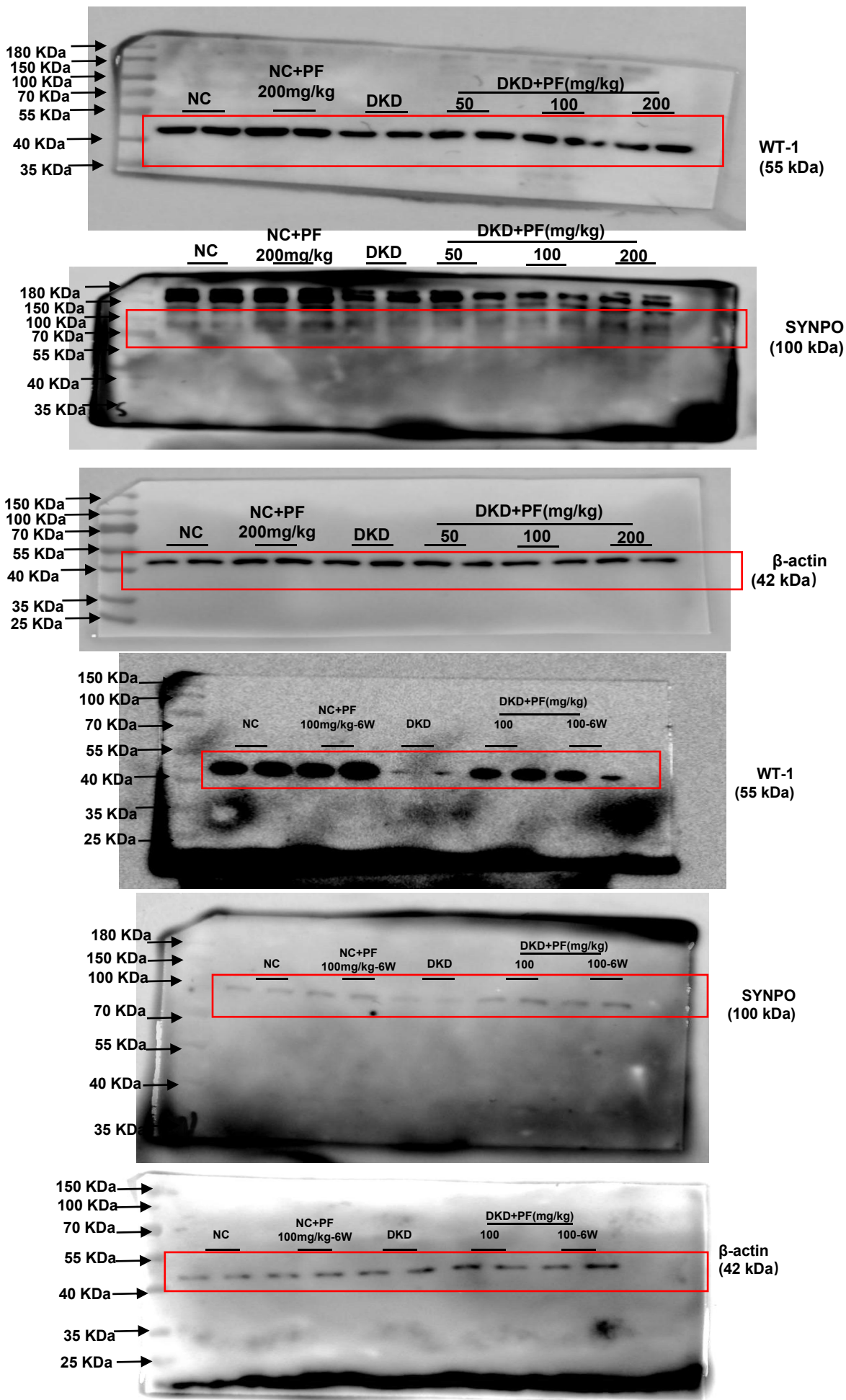

3

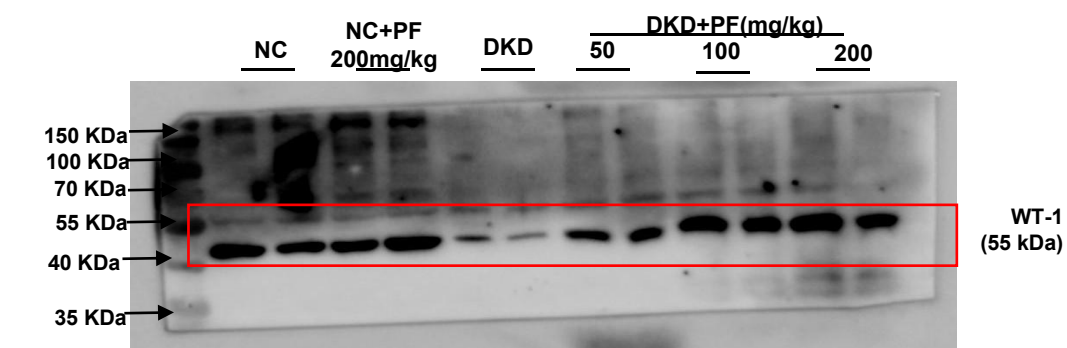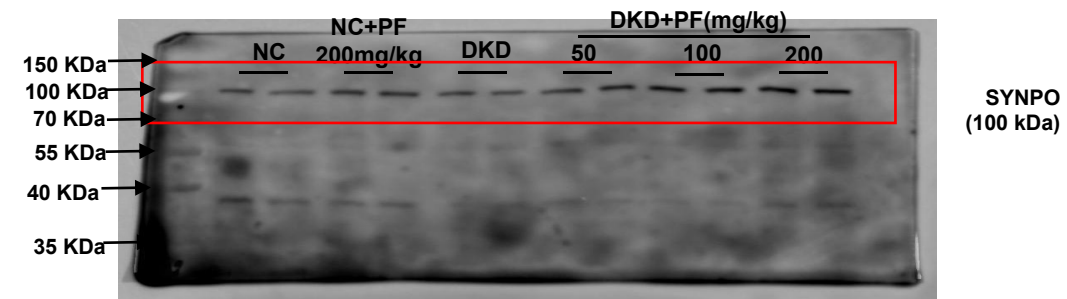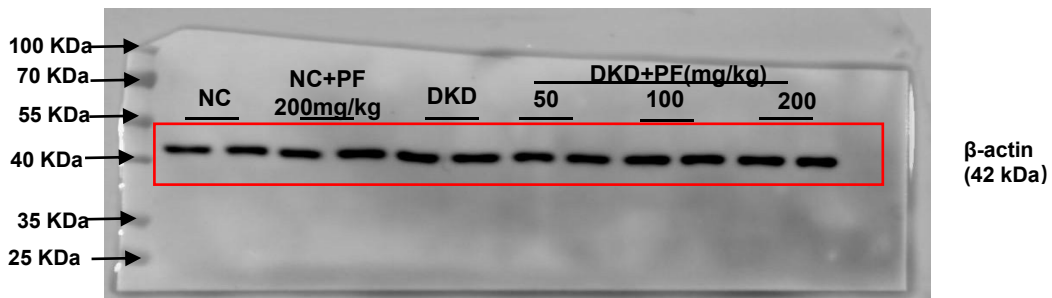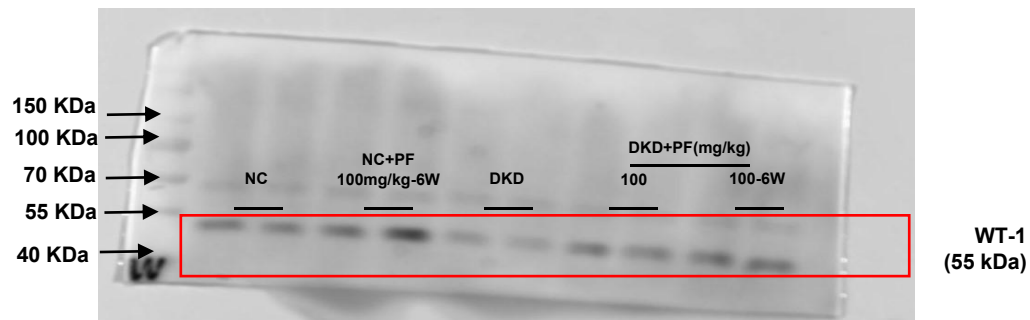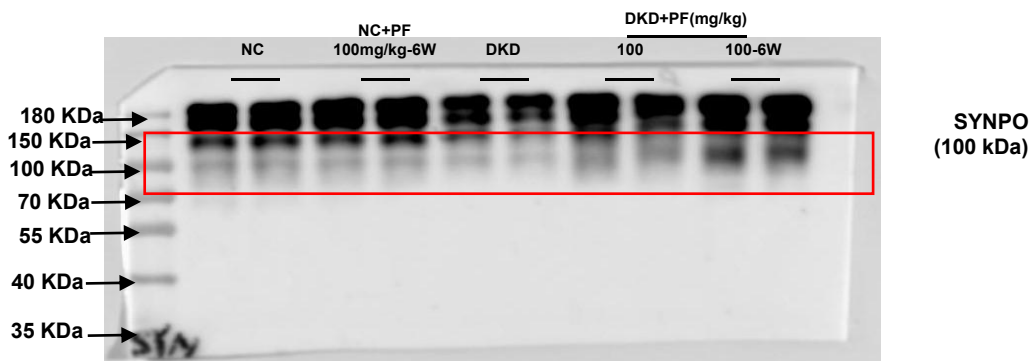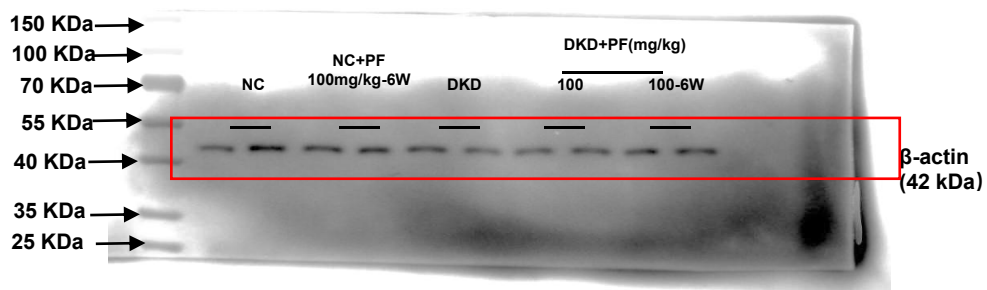

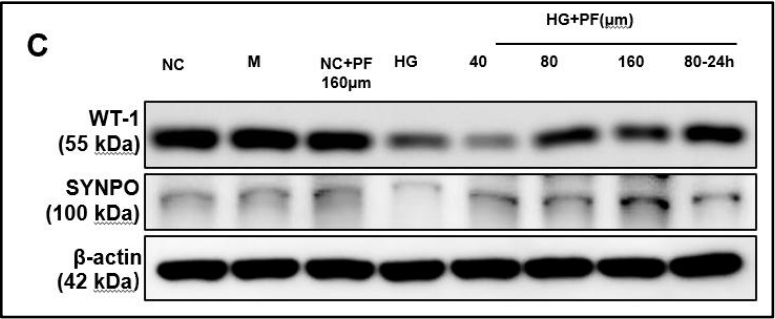

**1**

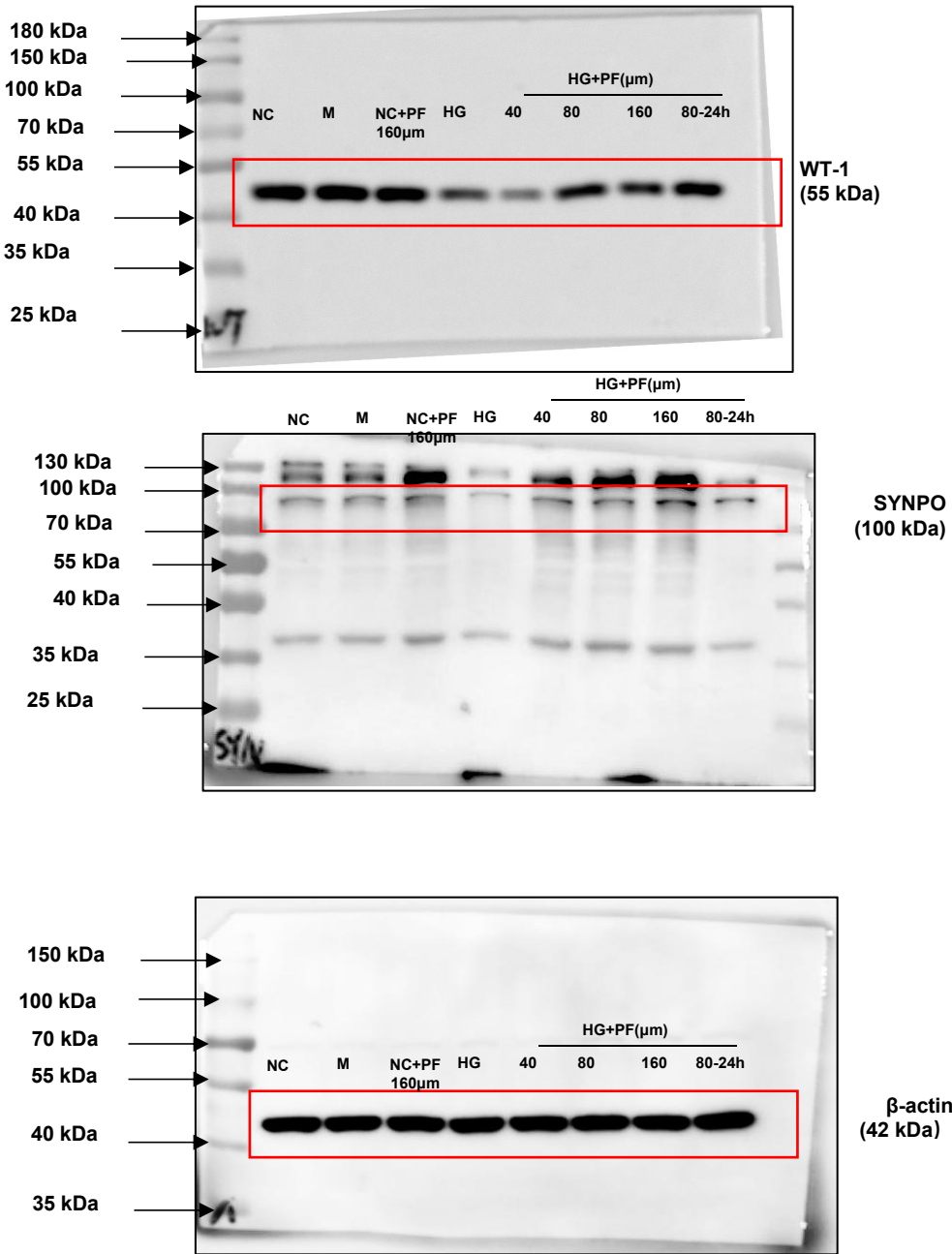

**Figure 5C**

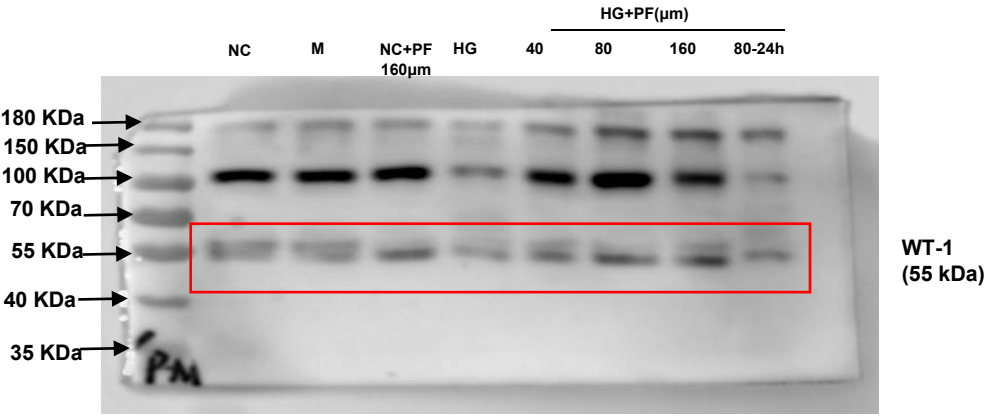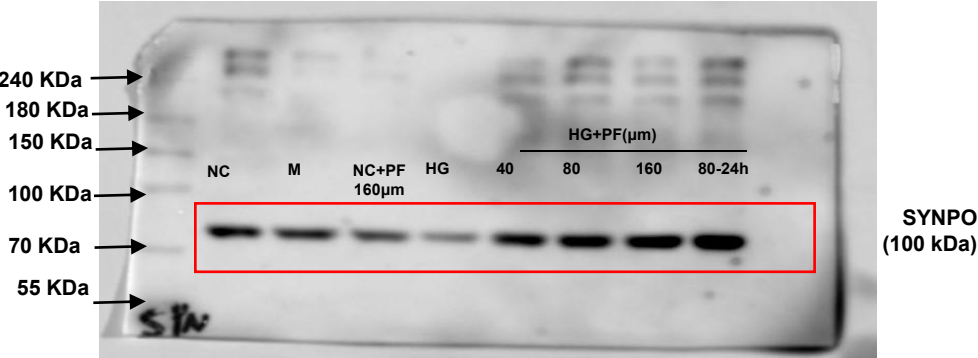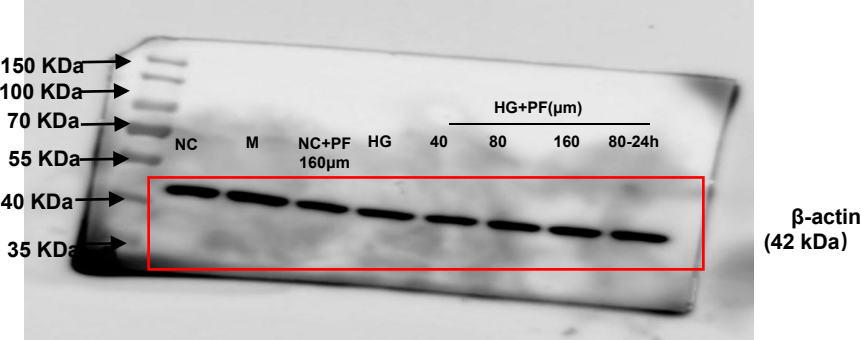

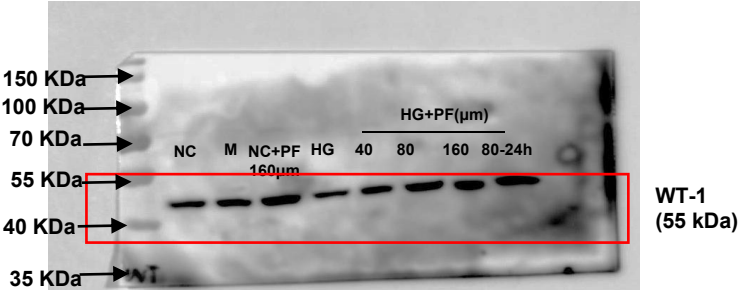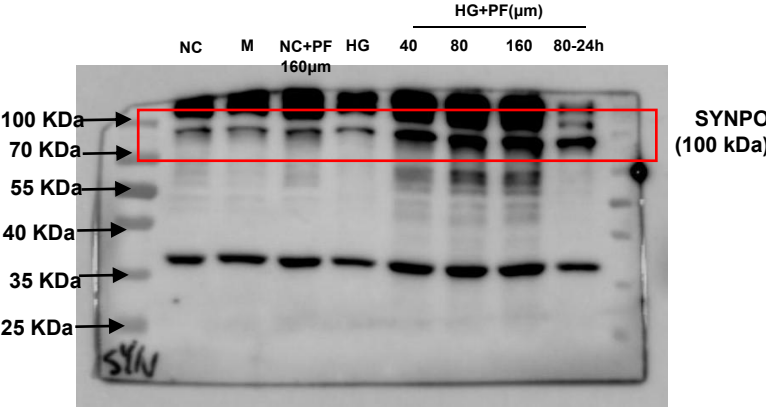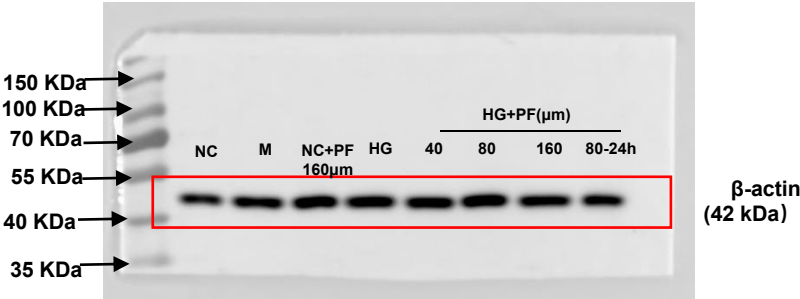



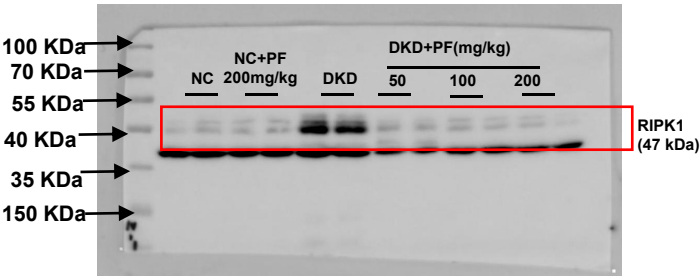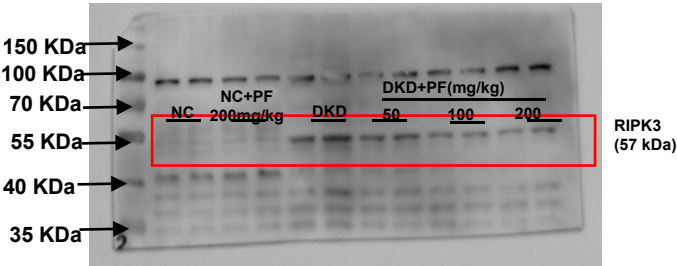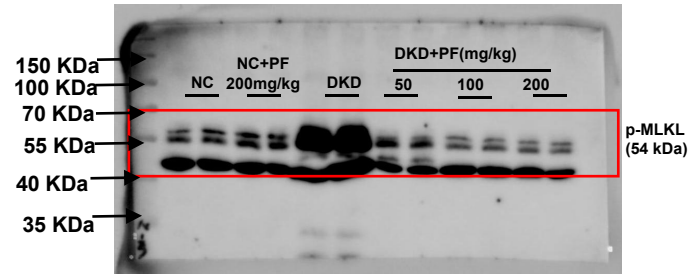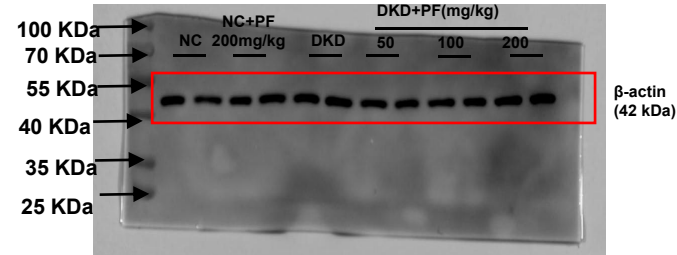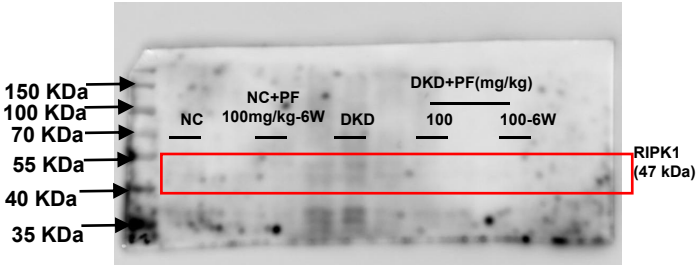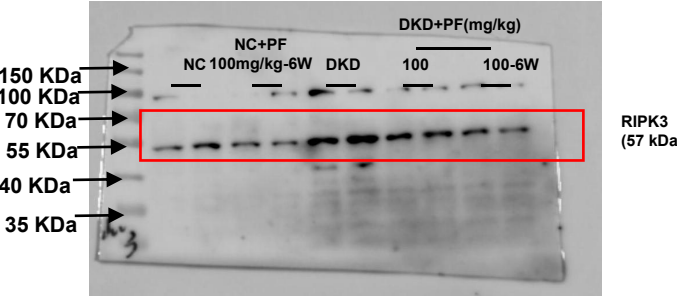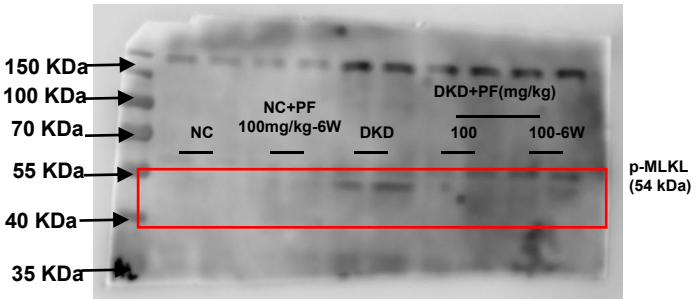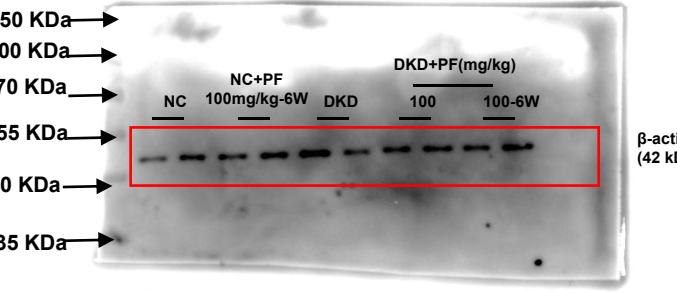

3

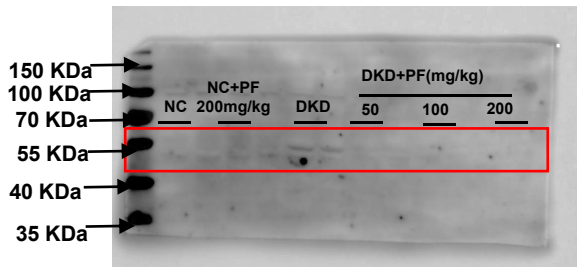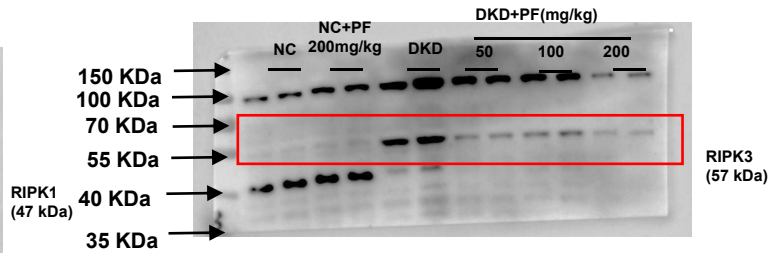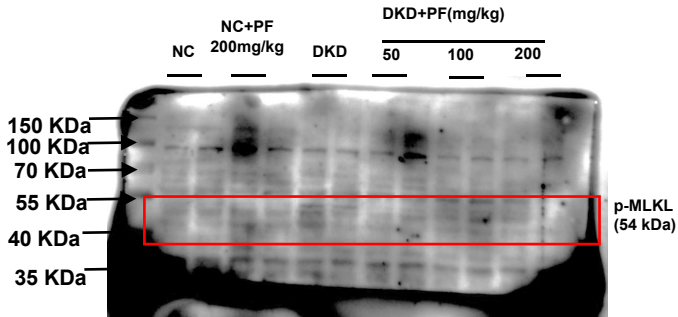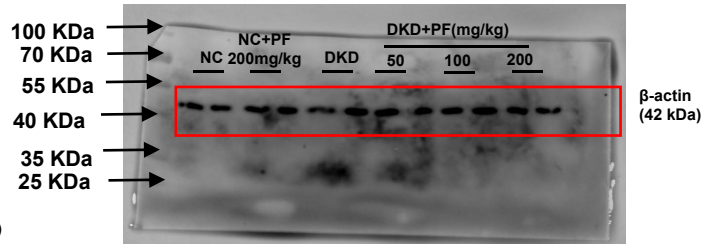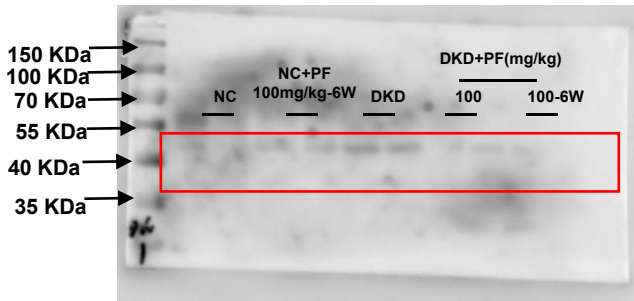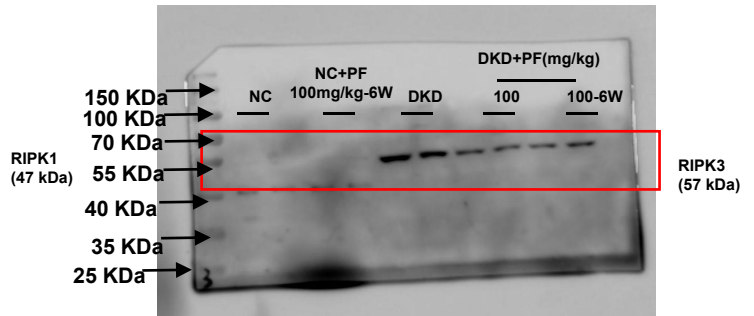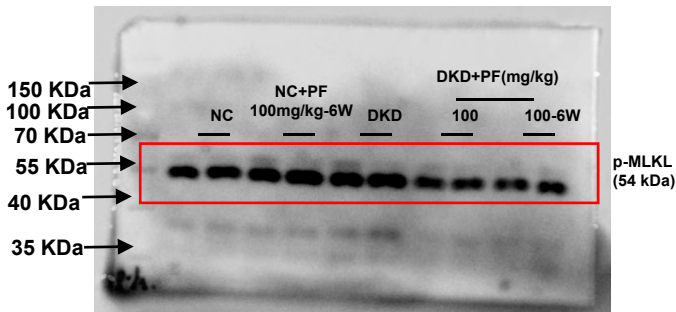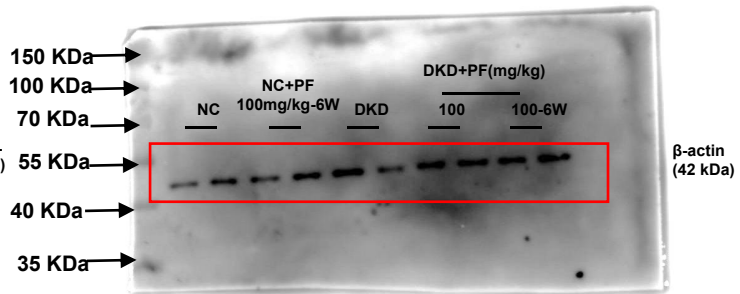

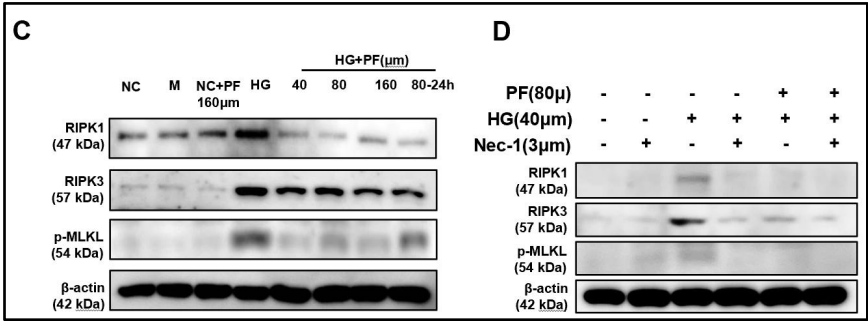

1

D

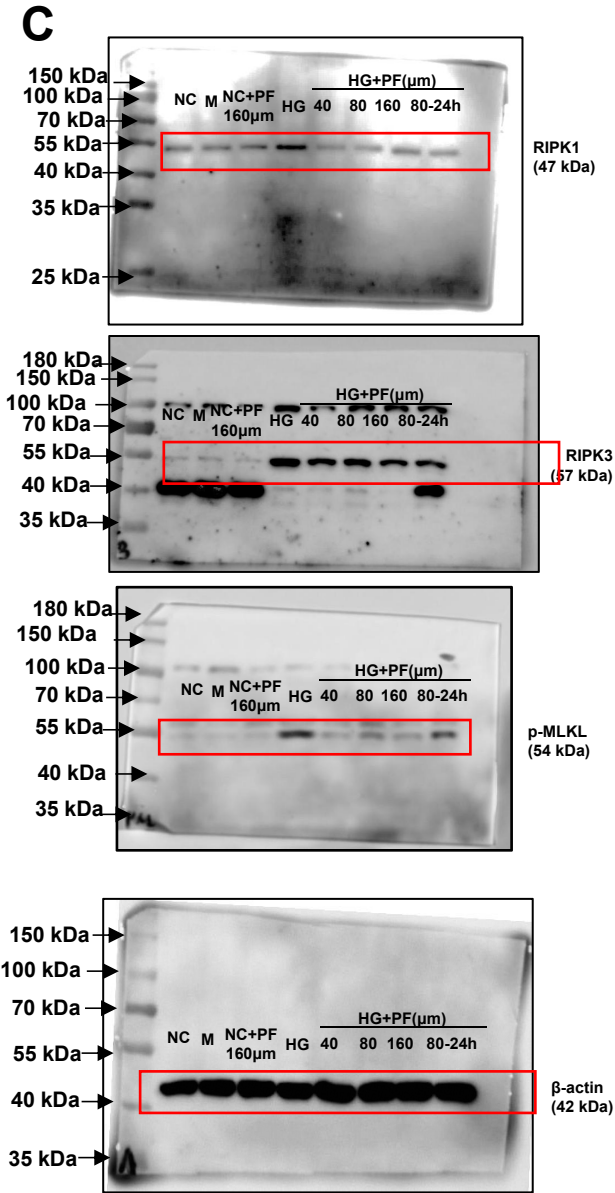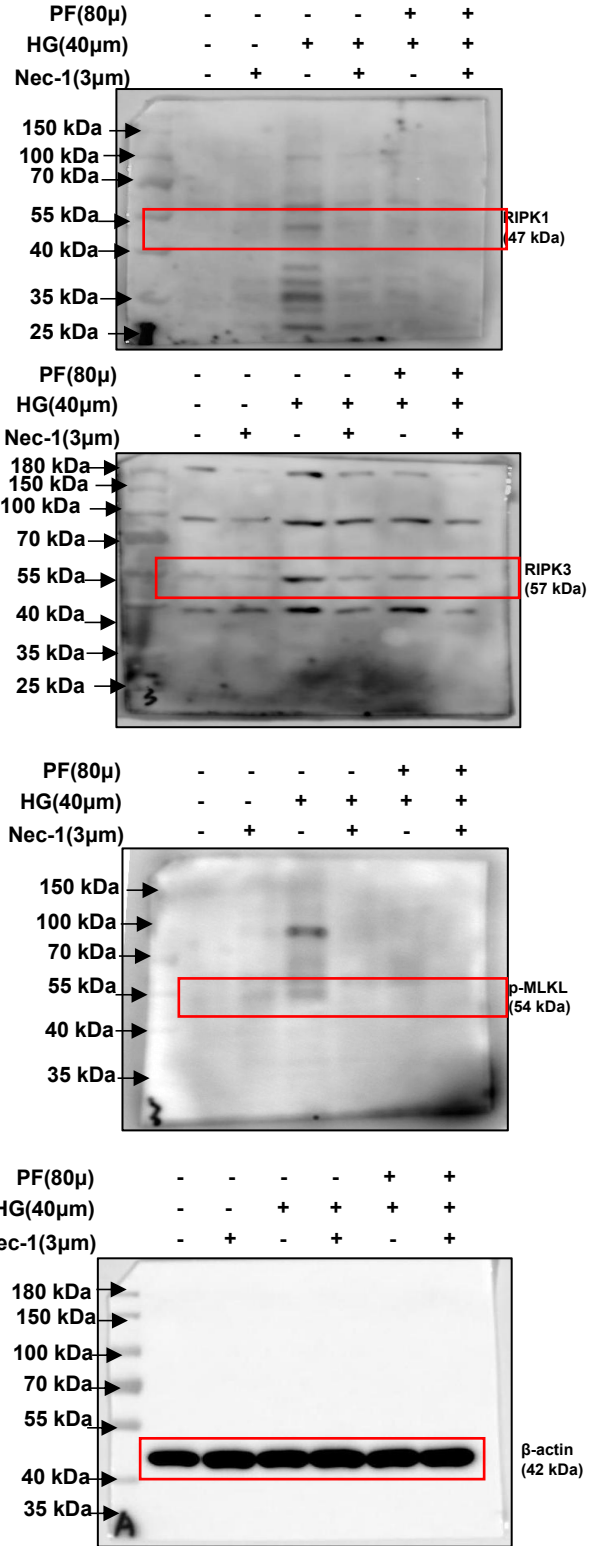

Figure 7C,D

2

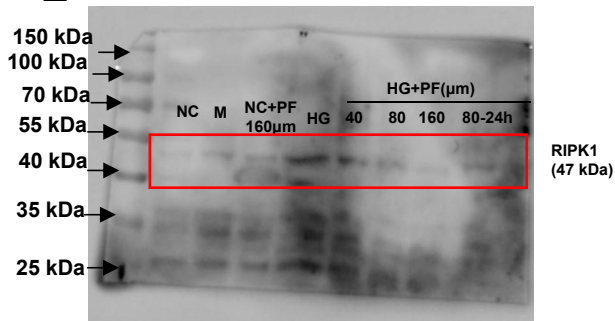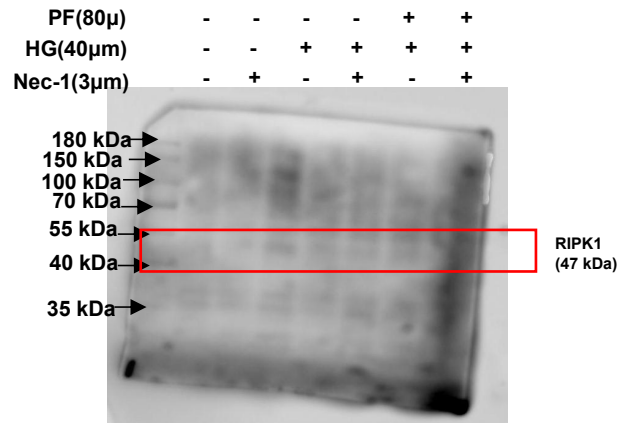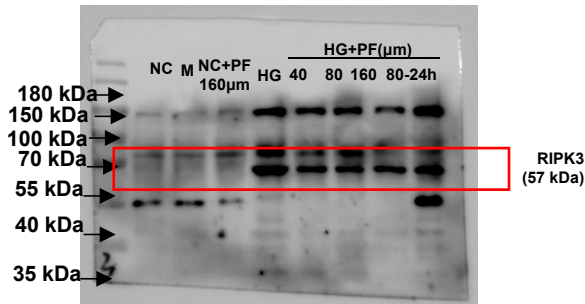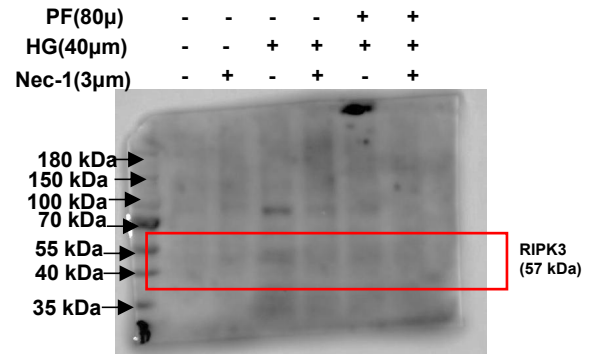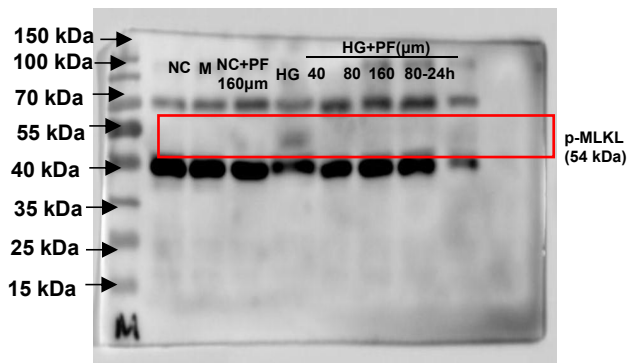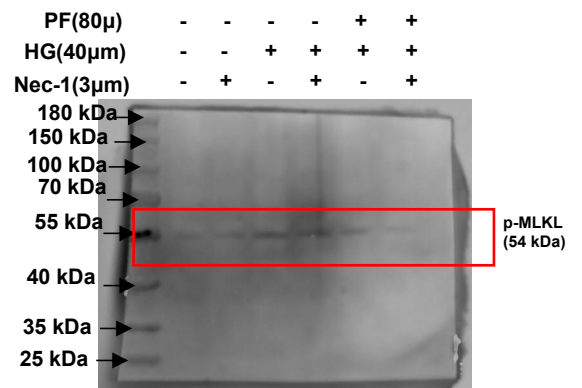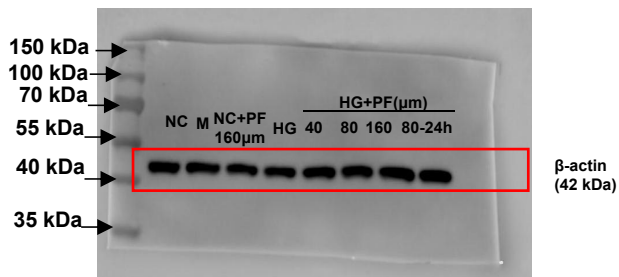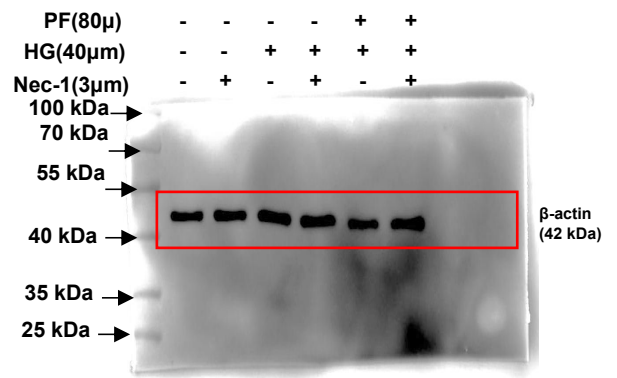

3

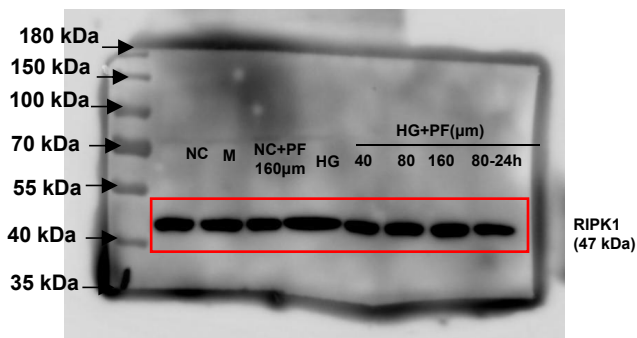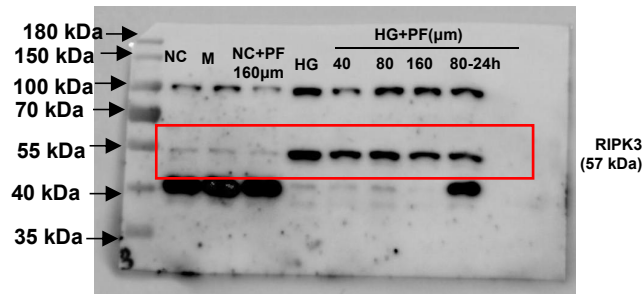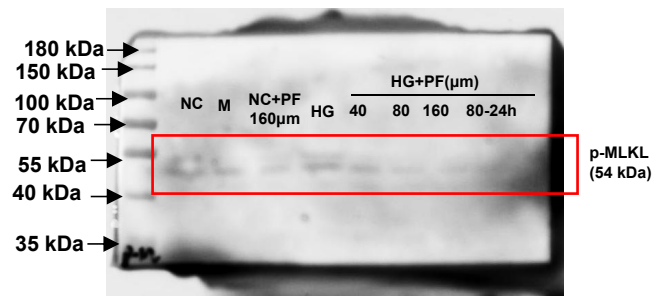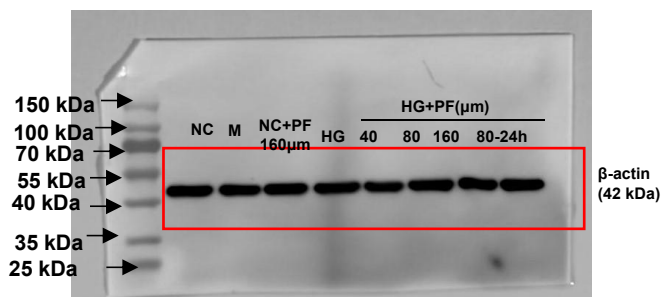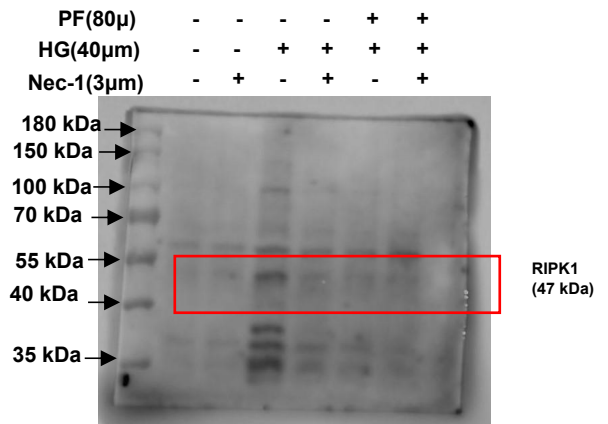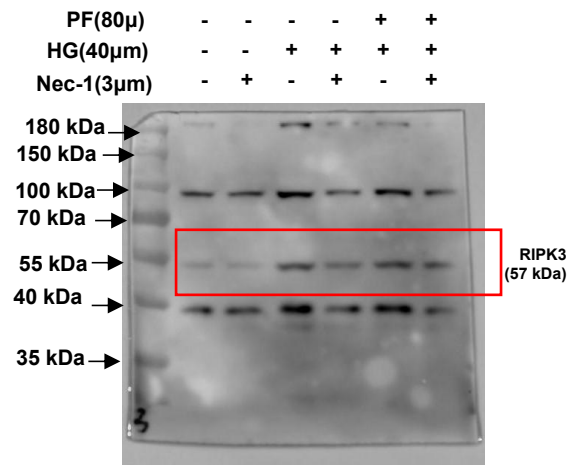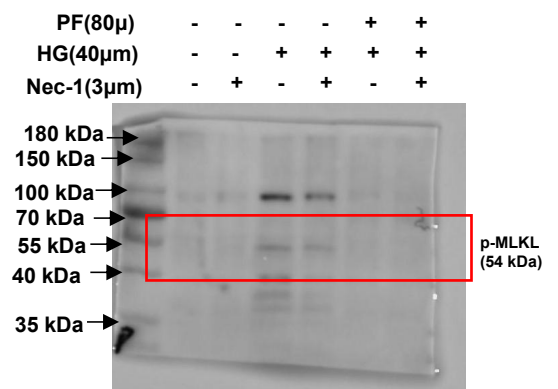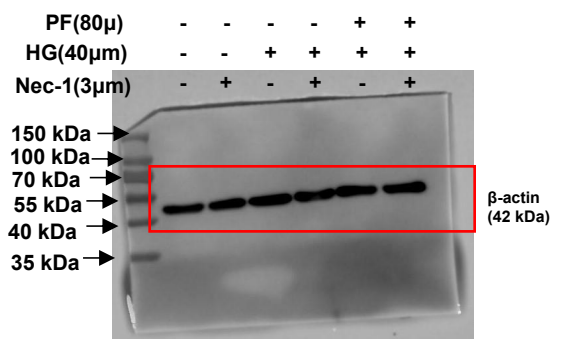

1

D

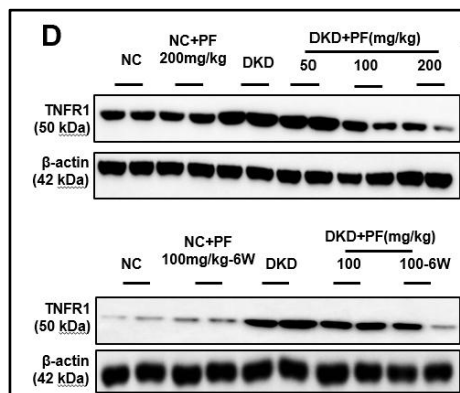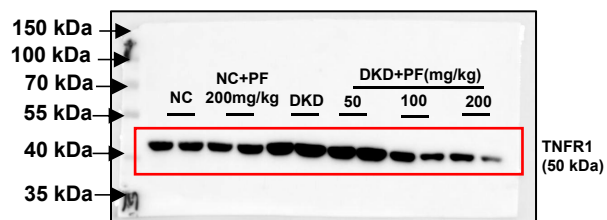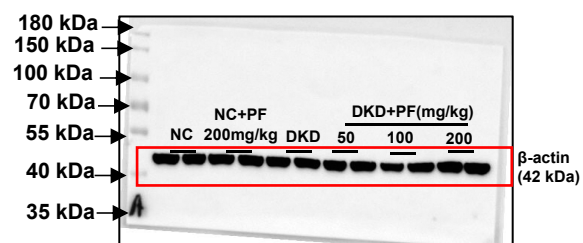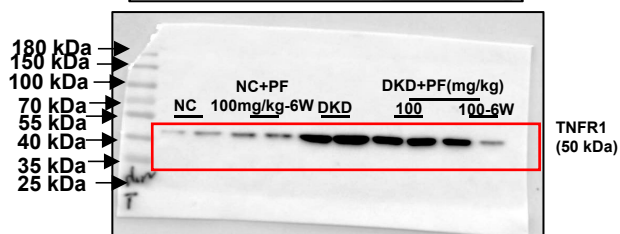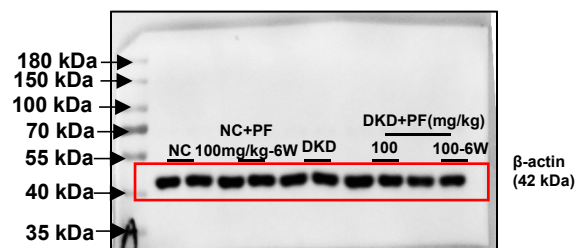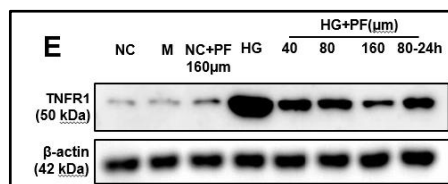

E

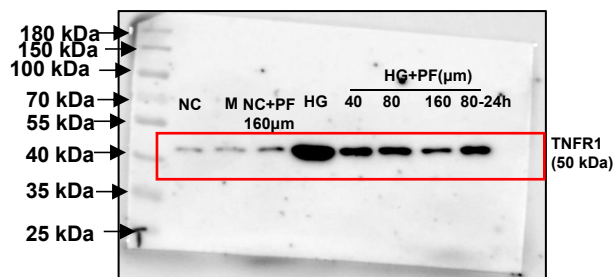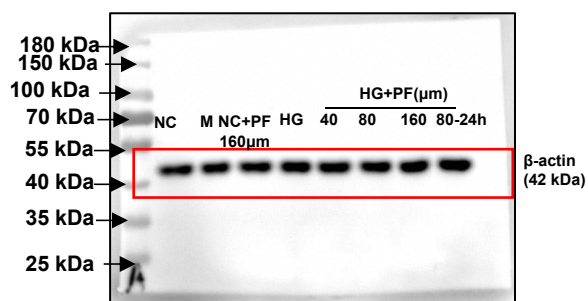

Figure 8D,E

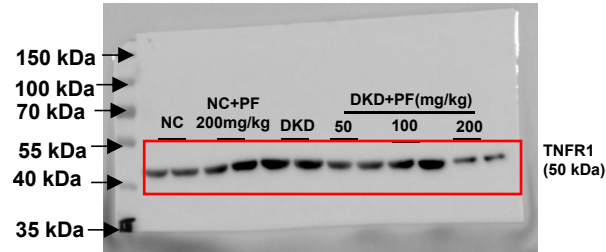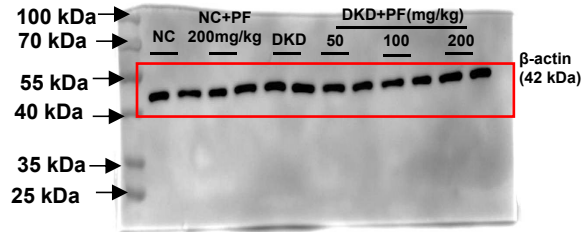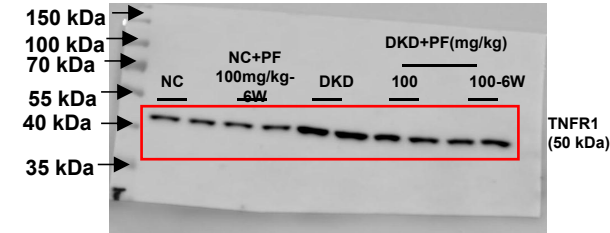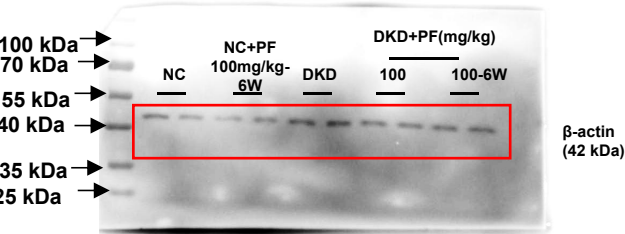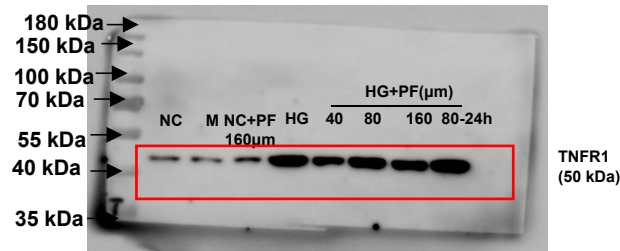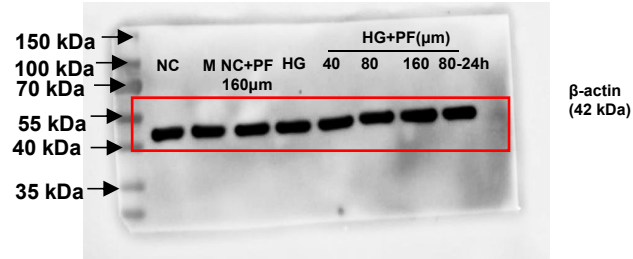

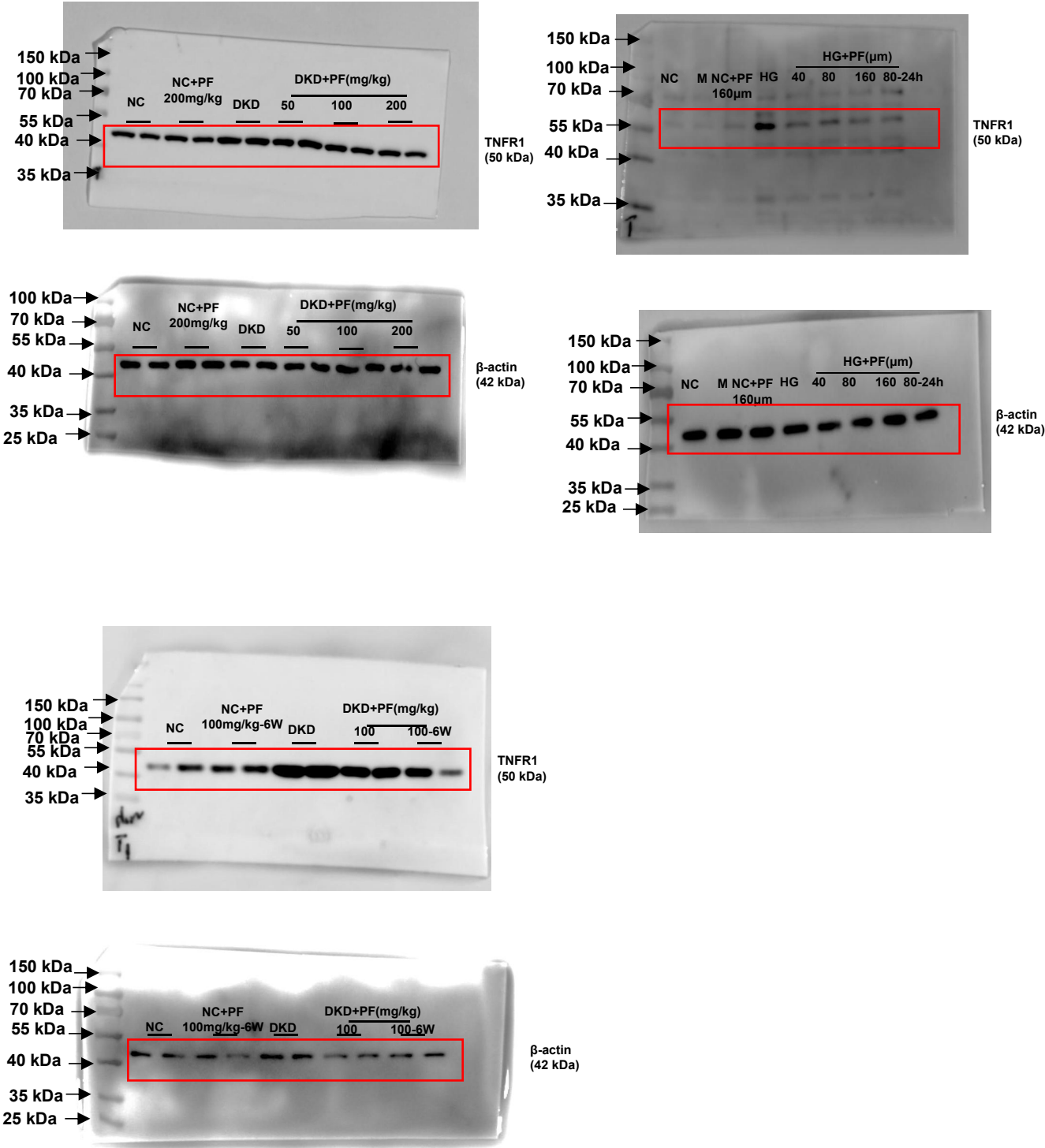

H

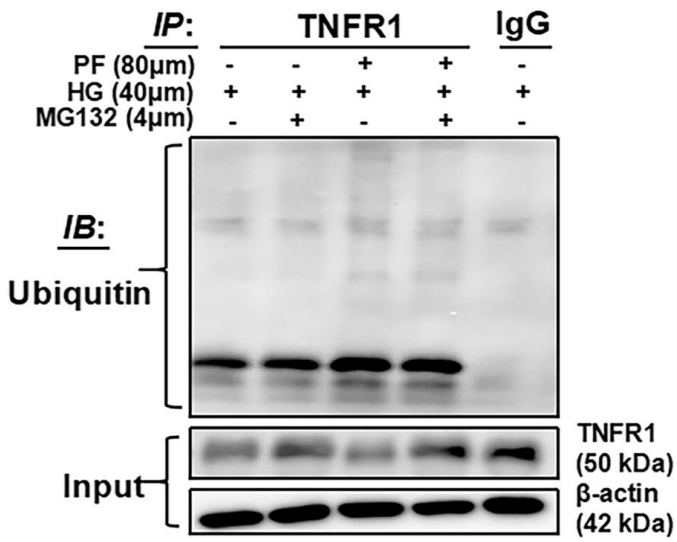

1

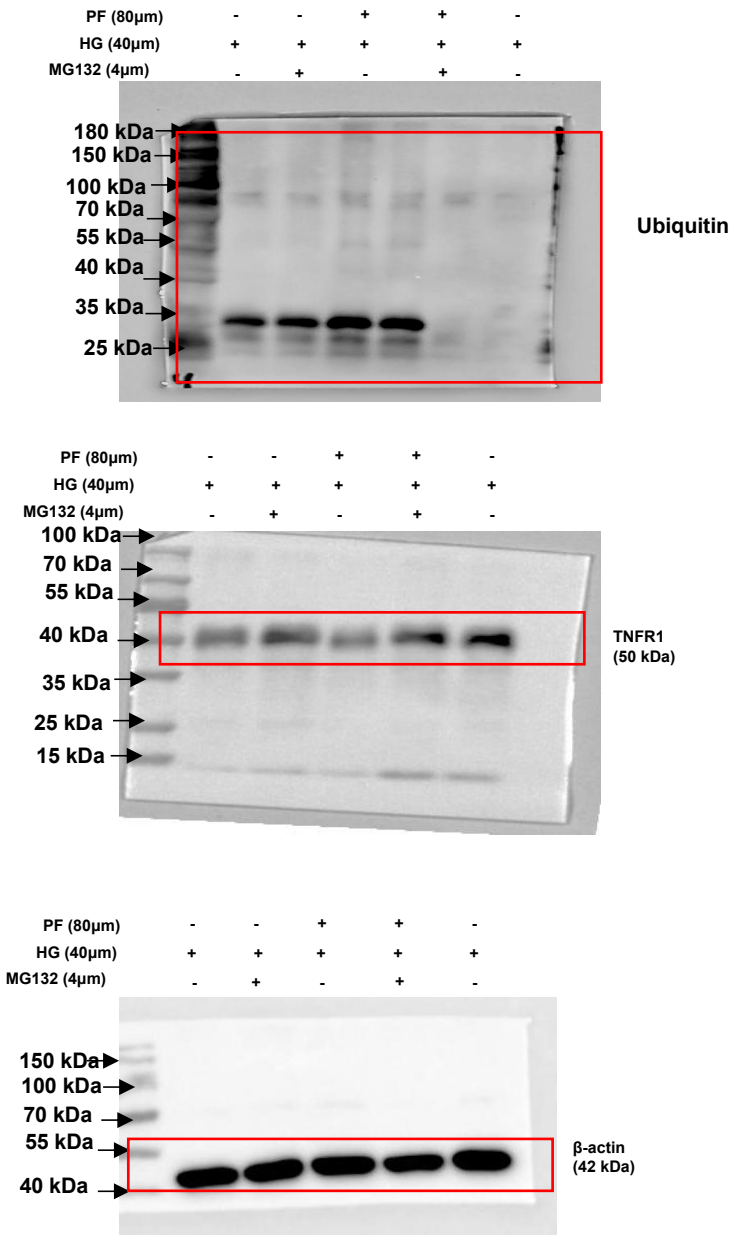

Figure 8H

2

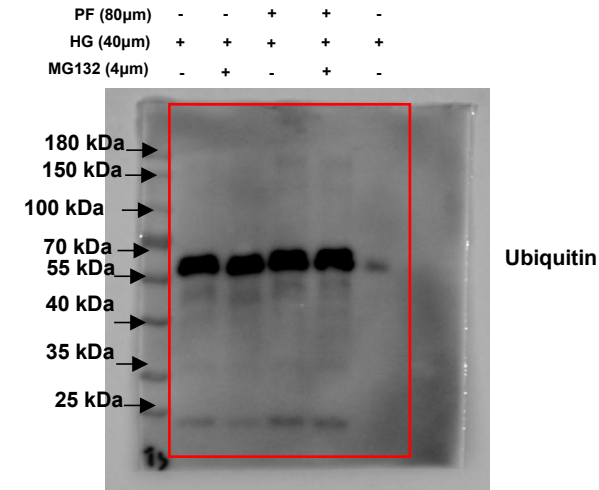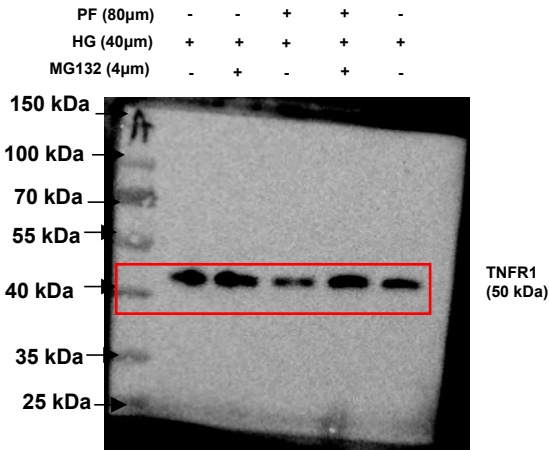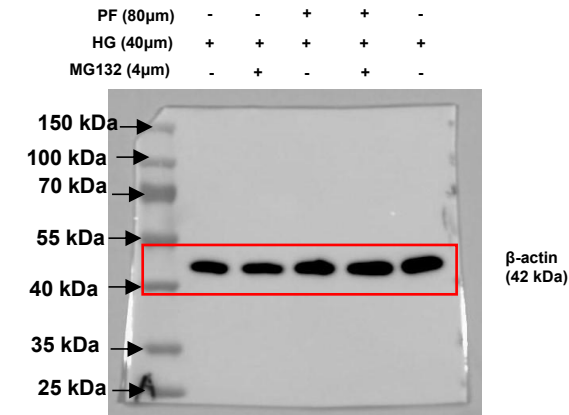

3

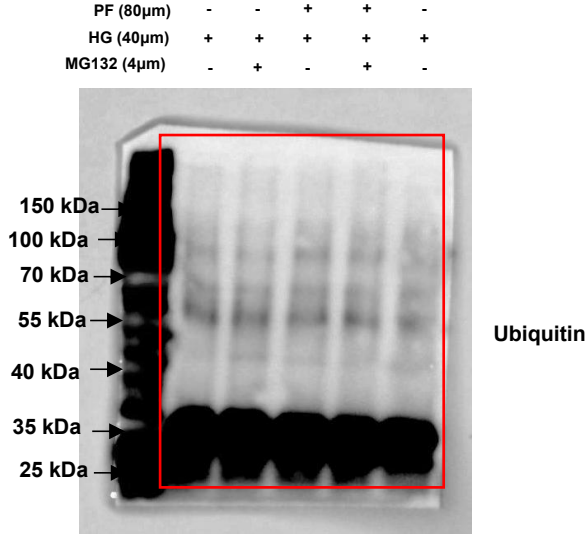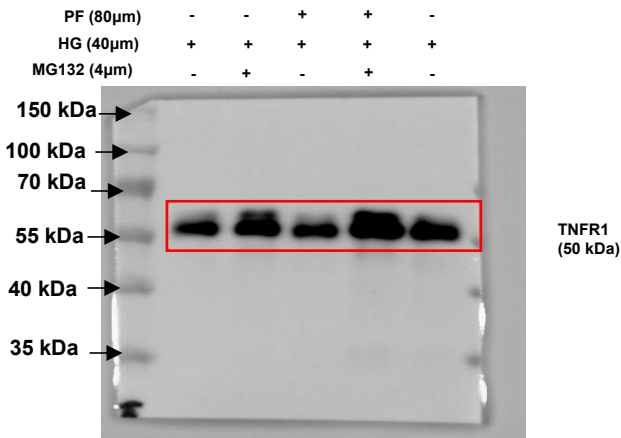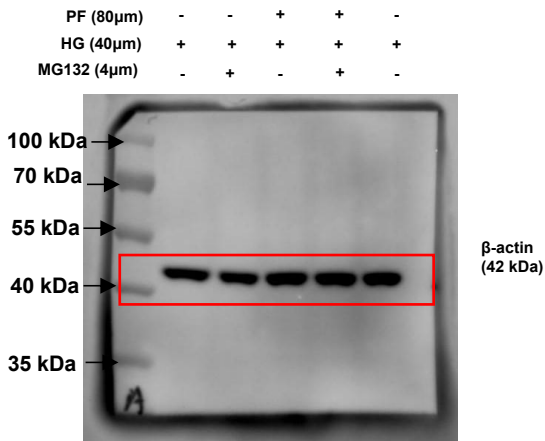

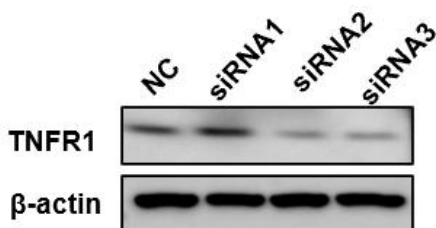

1

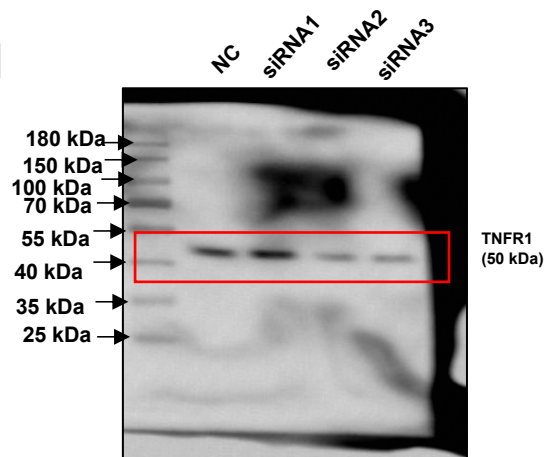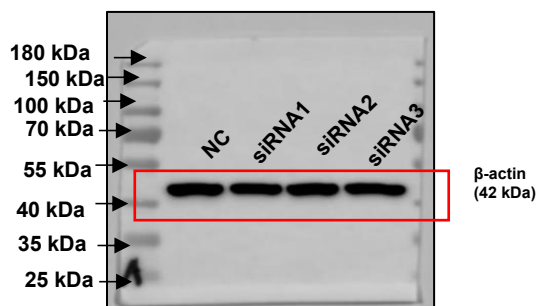

2

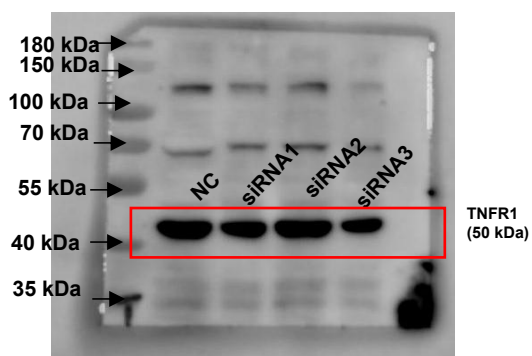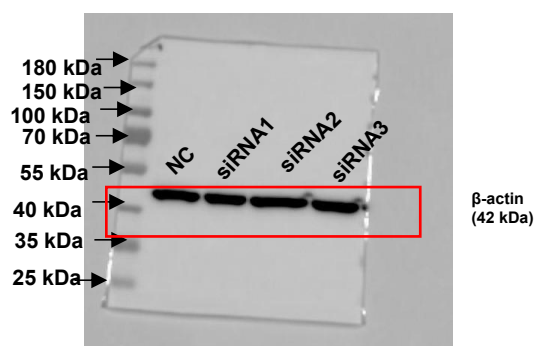

3

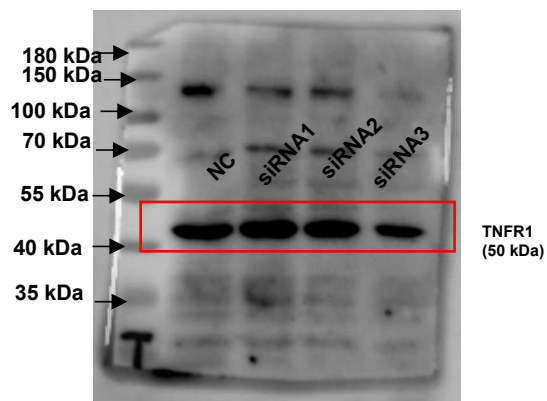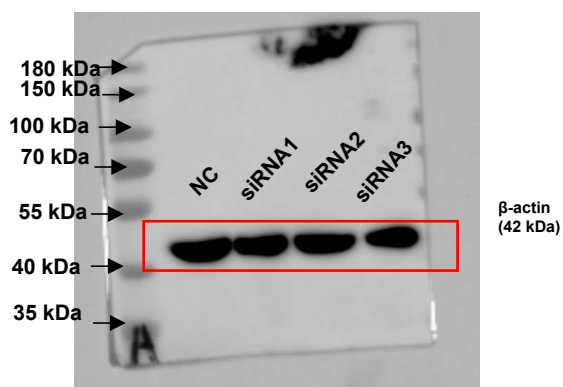

Figure 10B

1

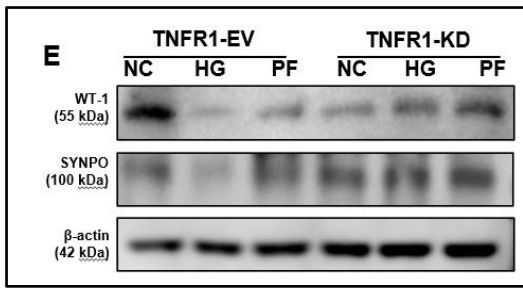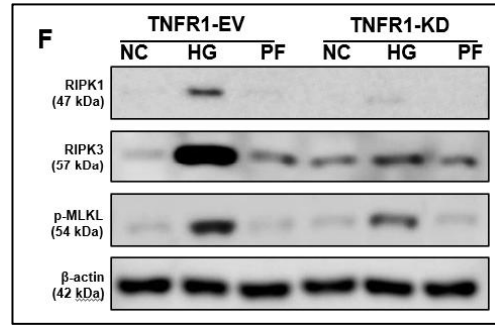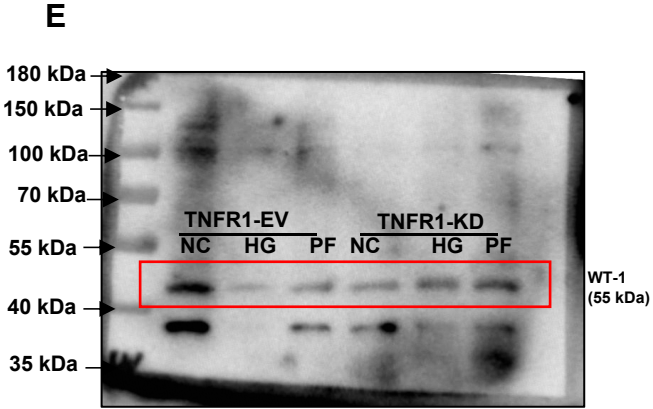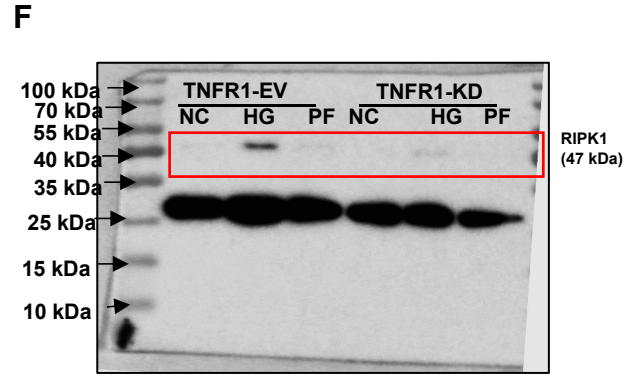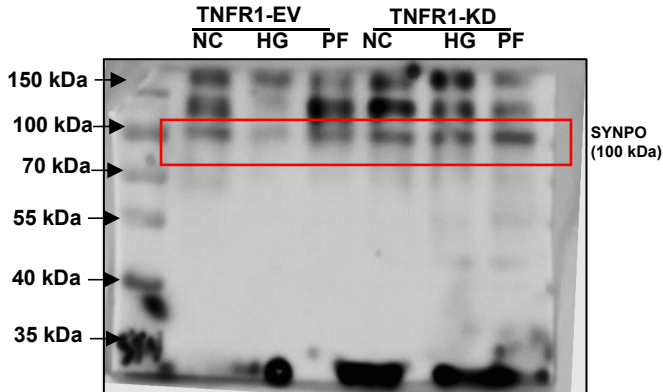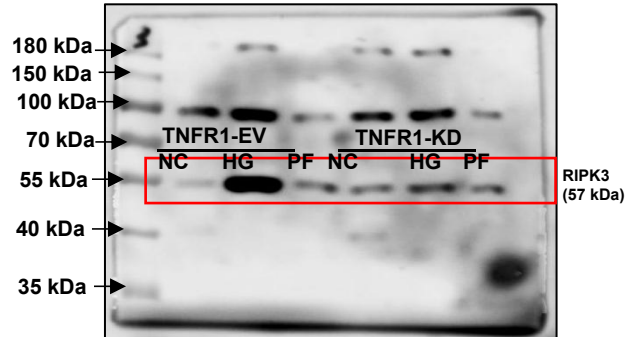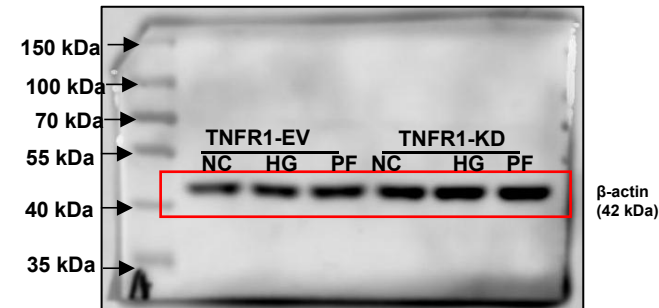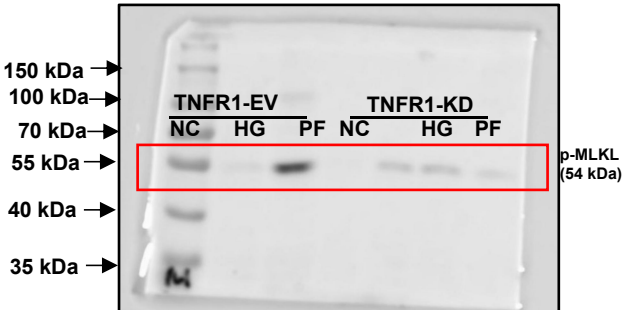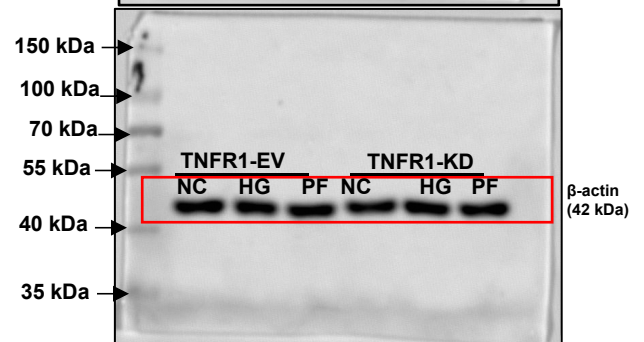

Figure 10E,F

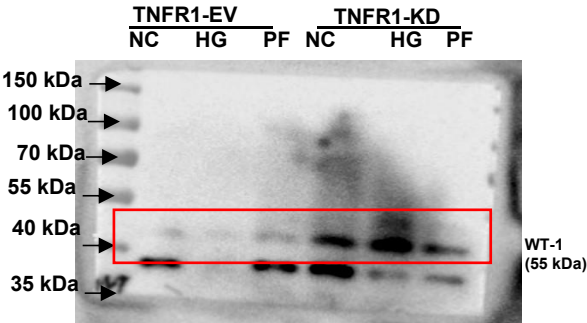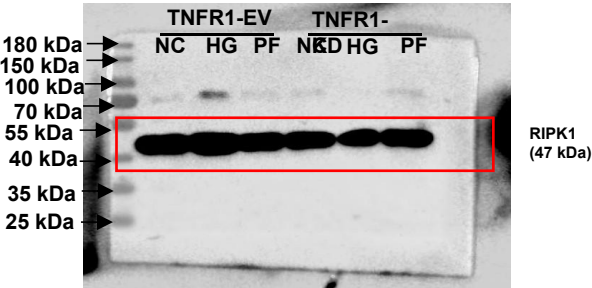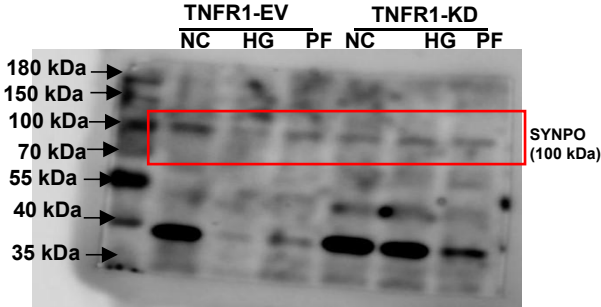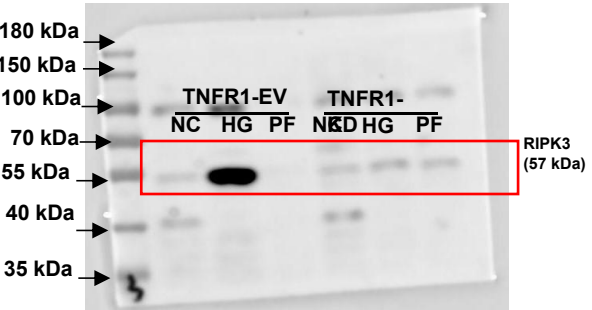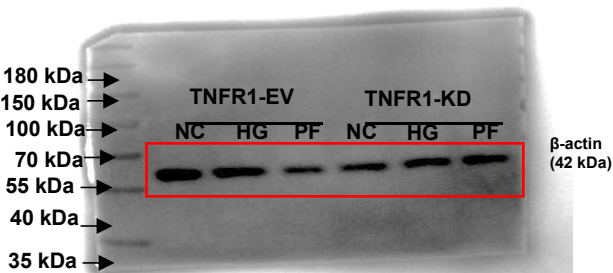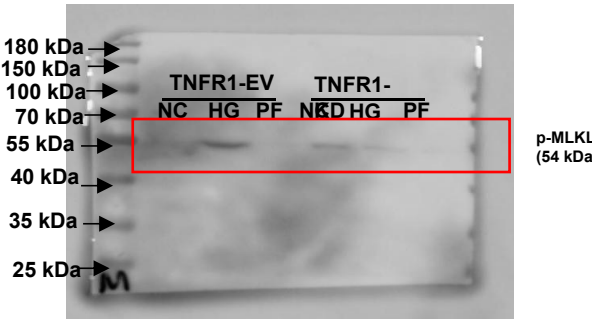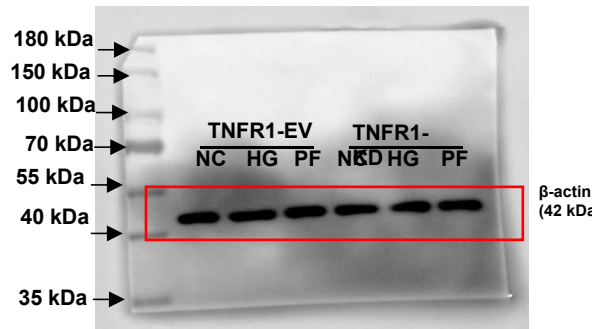

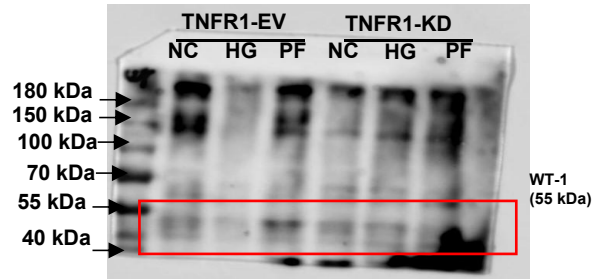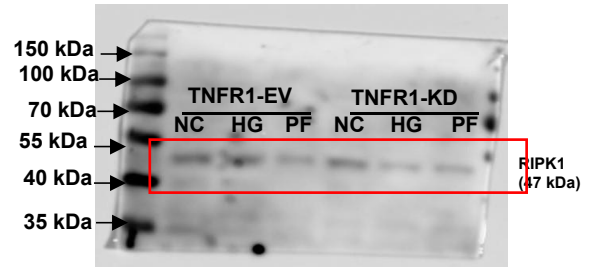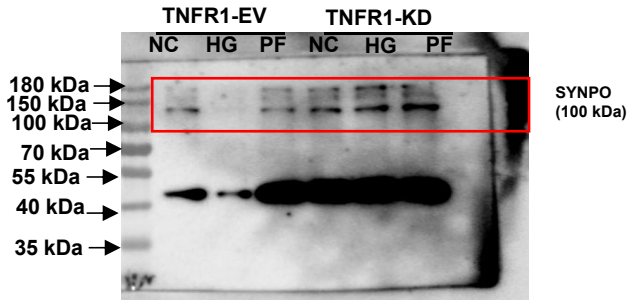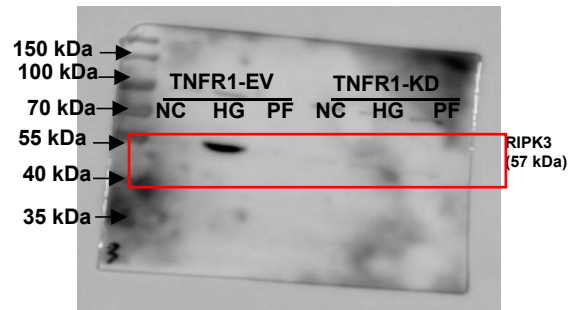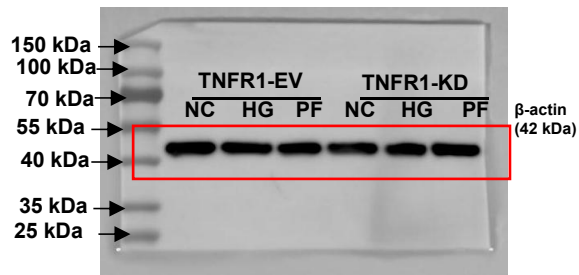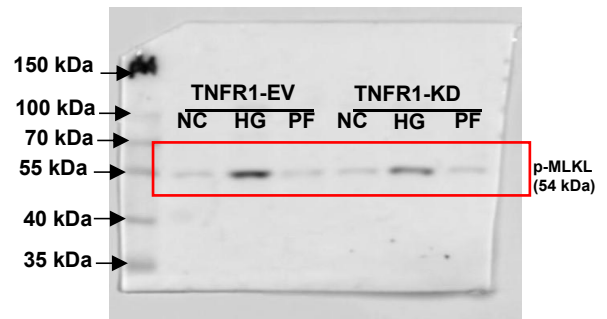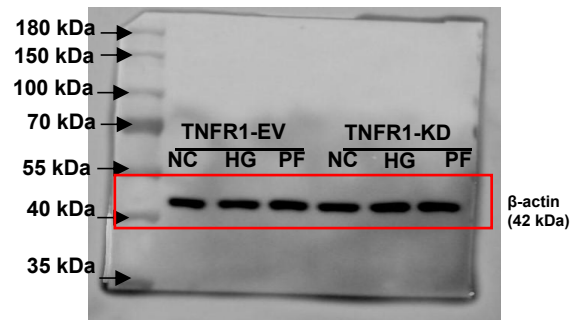

Supplement: Supplementary file 1 [file DataSheet1.PDF]

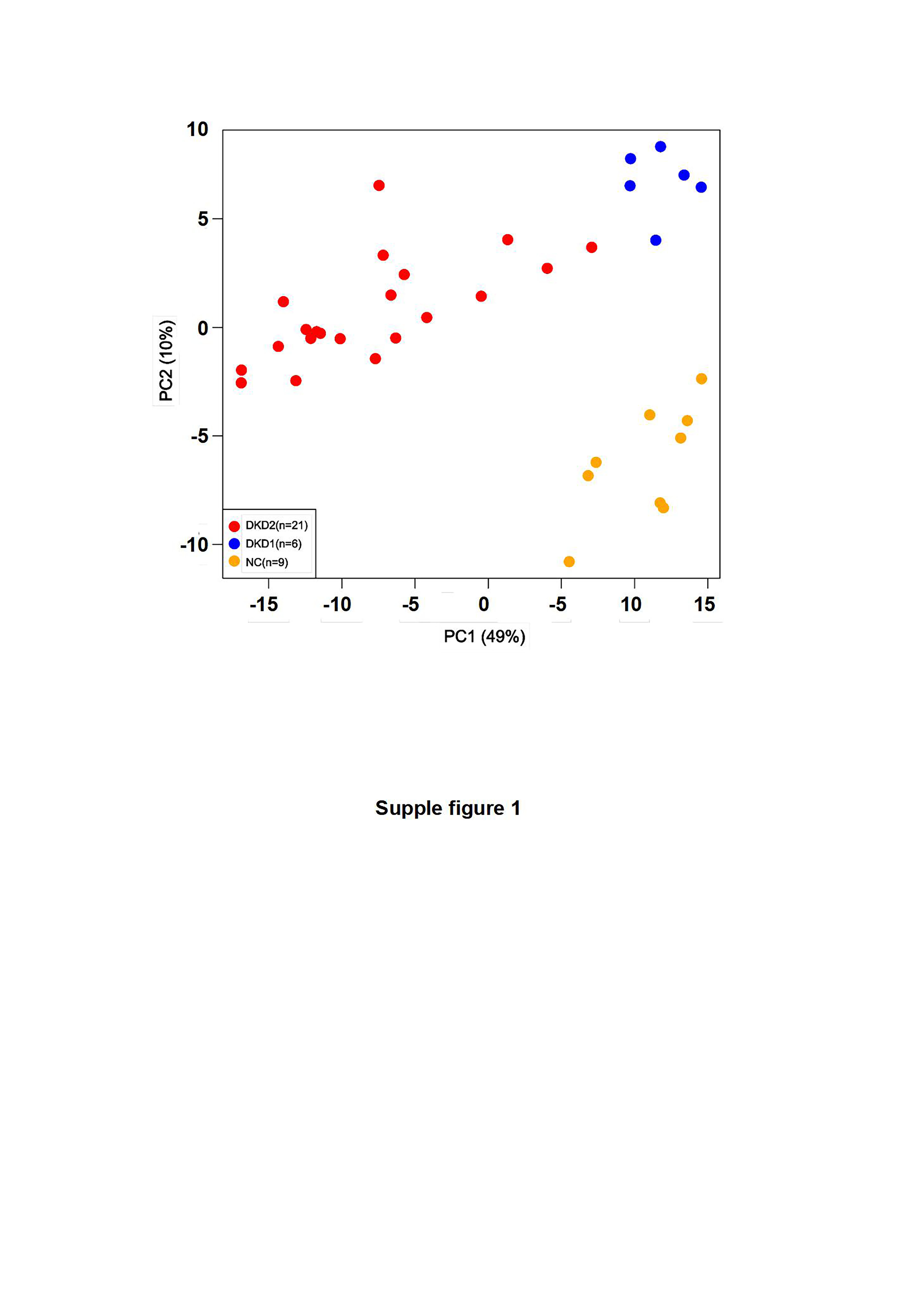

Supplement: Supplementary file 2 [file DataSheet2.ZIP › supple figure and table/Supple figure 1.jpg]

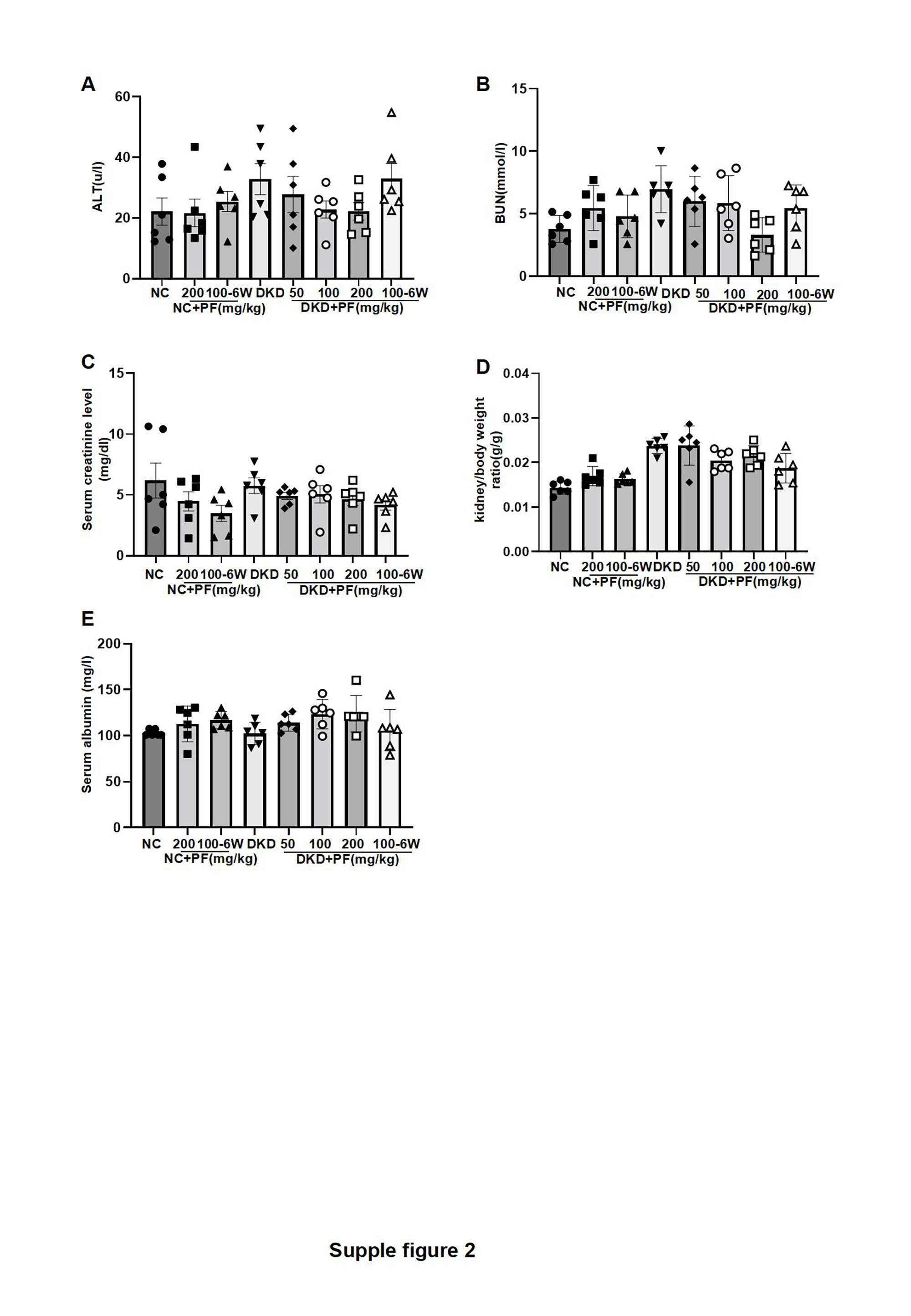

Supplement: Supplementary file 2 [file DataSheet2.ZIP › supple figure and table/supple figure 2.jpg]
