# Supplementary material for: Paeoniflorin directly binds to TNFR1 to regulate podocyte necroptosis in diabetic kidney disease
Source: Front Pharmacol. 2022 Sep 6;13:966645. doi: 10.3389/fphar.2022.966645 (PMC9486100; doi:10.3389/fphar.2022.966645)
Supplement: Supplementary file 2 [file DataSheet2.ZIP › supple figure and table/supple table 1.docx]

**Table S1.** Necroptosis-related to genes

| GO | Gene symbol |
| --- | --- |
| GO:0070266  necroptotic process | *Ripk1, Ripk3, Mlkl, Tnfrsf1A, Tp53, Arhgef2, Gzmb, Pygl, Casp2, Rbck1, Tlr3, Bok, Casp8, Trpm7, Fadd, Rnf31, Ipmk, Faslg, Cflar, Casp6, Cav1, Nlrp6, Slc25A4, Dnm1L, Pgam5, Fas, Zbp1, Birc3, Spata2, Birc2, Cyld* |
